# Pathways and signatures of mutagenesis at targeted DNA nicks

**DOI:** 10.1371/journal.pgen.1009329

**Published:** 2021-04-15

**Authors:** Yinbo Zhang, Luther Davis, Nancy Maizels

**Affiliations:** 1 Department of Immunology, University of Washington Medical School, Seattle, Washington, United States of America; 2 Department of Biochemistry, University of Washington Medical School, Seattle, Washington, United States of America; Duke University, UNITED STATES

## Abstract

Nicks are the most frequent form of DNA damage and a potential source of mutagenesis in human cells. By deep sequencing, we have identified factors and pathways that promote and limit mutagenic repair at a targeted nick in human cells. Mutations were distributed asymmetrically around the nick site. BRCA2 inhibited all categories of mutational events, including indels, SNVs and HDR. DNA2 and RPA promoted resection. DNA2 inhibited 1 bp deletions but contributed to longer deletions, as did REV7. POLQ stimulated SNVs. Parallel analysis of DSBs targeted to the same site identified similar roles for DNA2 and POLQ (but not REV7) in promoting deletions and for POLQ in stimulating SNVs. Insertions were infrequent at nicks, and most were 1 bp in length, as at DSBs. The translesion polymerase REV1 stimulated +1 insertions at one nick site but not another, illustrating the potential importance of sequence context in determining the outcome of mutagenic repair. These results highlight the potential for nicks to promote mutagenesis, especially in BRCA-deficient cells, and identify mutagenic signatures of DNA2, REV1, REV3, REV7 and POLQ.

## Author summary

A nick is the simplest form of DNA damage: a discontinuity in a single strand of the DNA backbone that does not change the chemical composition. Nicks are also the most frequent form of DNA damage, as they occur naturally in the course of transcription and DNA repair, and in response to ionizing radiation. Nicks were until recently ignored as a potential source of genomic instability. Here we use deep sequencing to show that mutations do occur at nicks and to identify those mutations and the factors that cause them.

## Introduction

Nicks are the most frequent form of DNA damage and a potential source of genomic instability in human cells. Nicks can initiate both mutagenesis and homology-directed repair (HDR; reviewed in [1]). Nicks occur naturally in the course of transcription and DNA repair, and they also result from chemical exposures and ionizing radiation (IR), which generates 100 nicks for every double-strand break (DSB; [2]). Nicks are significantly less mutagenic than DSBs [3–6] but nonetheless possess considerable potential to contribute to the overall burden of mutagenesis because of the frequency with which they occur. Nicks may pose a particular threat to genomic stability in tumors treated with IR, which has been a mainstay of cancer therapy for decades, and is currently used to treat over half of solid tumors.

Relatively little is known about pathways that promote mutagenesis at nicks. Evidence derived largely from reporter assays has shown that nicks are normally protected by RAD51, which is loaded onto DNA by BRCA2. Mutagenesis is stimulated in response to reduction in the abundance or activity of RAD51 or BRCA2 or factors that interact with BRCA2 to load RAD51 onto DNA; and by enhancing the activity of the anti-recombinogenic helicase RECQ5, which evicts RAD51 from single-stranded DNA [3,4,7]. Those same conditions stimulate recombination of nicks via an alternative HDR (a-HDR) pathway that uses single-stranded DNA as a donor [3,4]. These results suggest that, in the absence of protection provided by BRCA2/RAD51, single-stranded regions flanking nicks may become exposed and available to anneal with complementary DNA molecules or to undergo mutagenic repair. However, the factors and pathways that promote mutagenesis at nicks have not been identified.

To elucidate pathways of repair at nicks, we have characterized mutagenic events at targeted nicks in human cells by deep sequencing. Building on the very detailed current understanding of DSB repair, we explicitly asked whether factors that carry out DSB repair also act at nicks, in experiments designed to compare their roles at nicks and DSBs targeted to the same site on the CD44 gene. Strikingly, deep sequencing revealed that deletions and single-nucleotide variants (SNVs) are distributed asymmetrically at nicks. This asymmetry suggested that resection occurred predominately 5’ but also 3’ of the nick site, and depletion analysis identified a critical role for DNA2/RPA, which can resect with either 5’-3’ or 3’-5’ polarity [8–12]. Further analysis showed that DNA2 inhibited 1 bp deletions but contributed to longer deletions, as did REV7. POLQ played an unanticipated role in stimulating SNVs. Parallel analysis of DSBs identified a similar role for DNA2 and POLQ (but not REV7) in promoting deletions, and for POLQ in stimulating SNVs. Insertions were infrequent at nicks and most were 1 bp in length, as at DSBs. REV1, a translesion polymerase, stimulated +1C insertions at one nick site, but not another, evidence of the potential importance of sequence context in determining the outcome of mutagenic repair at nicks. These results highlight the potential for nicks to promote mutagenesis, especially in BRCA-deficient cells, define pathways of mutagenesis and identify mutagenic signatures of factors that carry out or regulate nick repair.

## Results

### BRCA2 depletion increases the frequency of mutations at nicks (but not DSBs)

**Fig 1:**
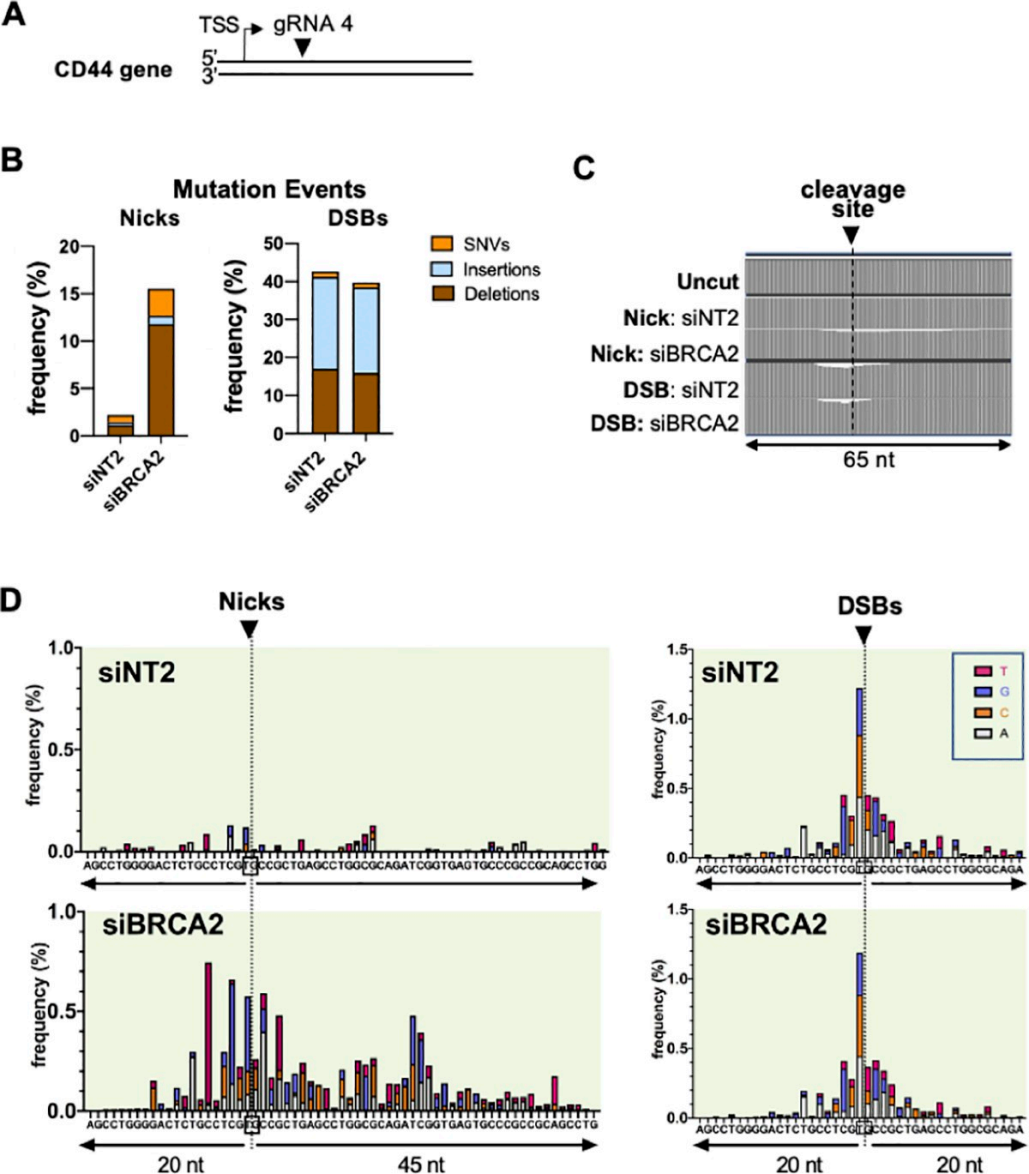
Depletion of BRCA2 increases the frequency of mutations at nicks. **(A)** Diagram of site targeted for cleavage in CD44 gene of human U2OS cells, showing transcription start site (TSS) and the site on the non-transcribed strand targeted for cleavage by gRNA 4 (arrowhead). **(B)** Frequencies of mutation events at nicks and DSBs targeted by gRNA 4, as determined by amplicon sequencing. Deletions, insertions and single nucleotide variations (SNVs) were scored in control cells (siNT2) or cells depleted for BRCA2. **(C)** Maps of the fractional decrease in the number of bases at each position within the indicated 65 nt region spanning the gRNA 4 cleavage site in U2OS cells. DNA was uncut, or targeted for nicks or DSBs, in control cells (siNT2) or cells depleted for BRCA2, as indicated. **(D)** Maps of positions and spectra of SNVs within the indicated regions spanning nicks or DSBs in U2OS cells treated with control siNT2 or siBRCA2. Arrowheads and dashed lines below them indicate the site of the nick or DSB, which targets the DNA backbone between the nucleotides boxed in the sequence below. The colored bar represents SNVs at each reference position: A, gray; C, orange; G, blue; T, red.

We examined frequencies of mutagenic events, including deletions, insertions and SNVs, at nicks and DSBs targeted to a site in the non-transcribed strand of the endogenous CD44 gene in human U2OS cells (**Fig 1A**). U2OS is a human osteosarcoma line that expresses wild-type P53 and that is frequently used to study DNA repair. CD44 encodes a cell-surface glycoprotein that is expressed in many cell types but not required for proliferation in cell culture. Surface CD44 is readily detected by antibody staining, making it possible to verify that observations document events at a transcribed gene by using flow cytometry to assess gene expression. DNA nicks or DSBs were targeted to exon 1 of the CD44 gene by Cas9D10A or Cas9, respectively, and CRISPR gRNA 4. Genomic DNA was isolated from populations of 10,000–30,000 transfected cells, PCR-amplified, and deletions, insertions and SNVs were scored within a 65 bp window by amplicon sequencing on an Illumina platform. Control experiments analyzing uncleaved DNA established the combined error frequencies of PCR amplification, sequencing, and computational analysis. Indels and SNVs were assessed using a computational strategy that greatly reduced PCR and sequencing errors by making a consensus call for groups of DNA molecules that contained the same unique molecular identifiers (UMI; see Methods). Absolute frequencies of indels and SNVs varied among experiments, but the effects of depletion of specific factors were reproducible. Results are presented for representative sequenced libraries.

The frequency of mutagenic events was considerably lower at nicks than at DSBs, as illustrated by the representative analysis in **Fig 1B** (2.2% vs. 42%; 19-fold). At nicks, most mutagenic events were deletions, with fewer SNVs and few insertions; while at DSBs most were insertions, with somewhat fewer deletions and relatively few SNVs. Depletion of BRCA2 increased the frequency of mutagenic events at nicks 7-fold (p <0.01), but had no significant effect at DSBs (p = 0.985).

Maps of the fractional decrease in the number of base calls at each position showed that, in cells depleted of BRCA2, deletions exhibited an asymmetric distribution with respect to the nick site, extending from approximately 10 bp 5’ (upstream) to 25 bp 3’ (downstream); while at DSBs, deletions were clustered within approximately 10 bp on either side of the cleavage site (**Fig 1C**). SNVs also exhibited asymmetric distribution around the target site of nicks, but not of DSBs (**Fig 1D**). The asymmetry at nicks was especially clear in the map of SNVs in BRCA2-depleted cells (**Fig 1D** left). In contrast, at DSBs SNVs were clustered around the target site, in a pattern relatively unaffected by BRCA2 depletion (**Fig 1D** right). Insertions proved to cluster at the target site, as discussed below.

### Asymmetry of mutations at nicks does not reflect the direction of transcription

The asymmetric distribution of mutations at nicks could reflect activity of a pathway in which nicks undergo predominately 5’ but also 3’ resection; or it could depend upon the direction of transcription. To address the latter possibility, we mapped mutations targeted by gRNA 7 to a site located 35 bp upstream of the gRNA 4 site and on the opposite DNA strand (**S1A and S1B Fig**). If resection is predominately 5’ at both nicks, more events should be evident in the zones flanking the nick sites than the zone between the nick sites (**S2A Fig**). This was tested among mutants generated within the same cell population by co-transfecting cells with RNPs composed of Cas9D10A and either gRNA 4 or gRNA 7 at RNP levels titrated to prevent dual cleavage. Sequencing showed that in >99% of molecules that bore evidence of mutation, mutagenic events were restricted to a single site. Graphs of frequencies of mutations (including deletions, SNVs and insertions) identified a single asymmetric peak at nicks in populations transfected with RNPs containing gRNA 4/Cas9D10A, and a single asymmetric peak at DSBs in populations transfected with RNPs containing gRNA 4/Cas9 (**S2B** and **S2C Fig**). Two peaks were evident in populations co-transfected with RNPs composed of Cas9D10A and either gRNA 4 or gRNA 7. The mutation frequency was sufficiently high in the BRCA2-depleted sample to make it evident that asymmetry at one site was the mirror image of asymmetry at the other (**S2D Fig).** Thus asymmetry does not simply reflect transcriptional orientation.

### DNA2 and RPA1 promote resection resulting in a-HDR or mutagenesis

**Fig 2:**
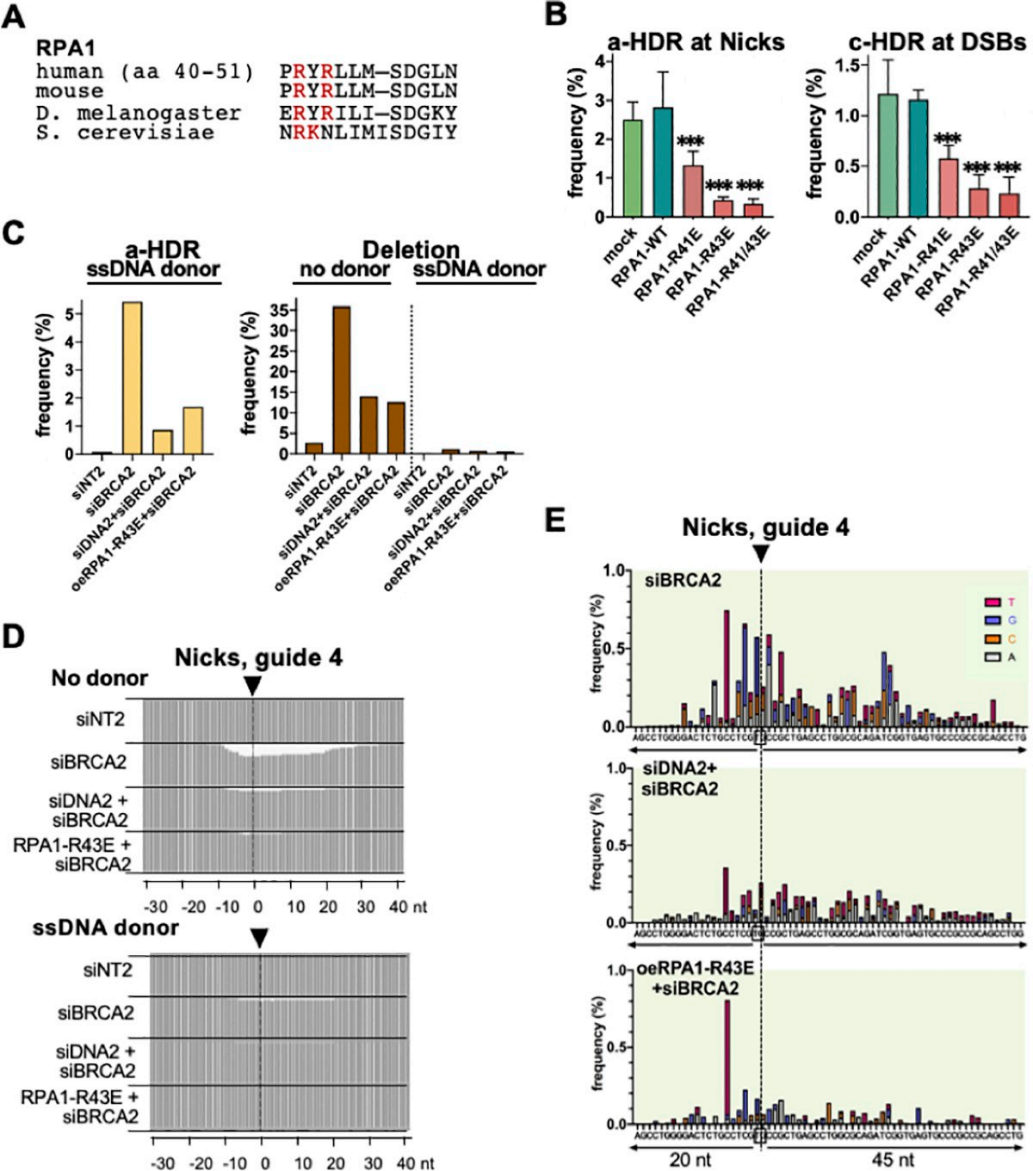
DNA2 and RPA1 promote resection resulting in a-HDR or mutagenesis at nicks. **(A)** Alignment of RPA1 protein sequences in the conserved N-terminal region to which the *S*. *cerevisiae* Rfa1-t11 (K45E) mutation maps [22]. **(B)** Frequencies of a-HDR at nicks or c-HDR at DSBs as determined by reporter assays in 293 TL cells ectopically expressing RPA1 WT or its mutant derivatives. Frequency values represent the mean ± SEM from a representative experiment; *** indicates p<0.001 for the frequency difference between the indicated sample and sample expressing RPA1-WT. **(C)** Frequencies of a-HDR at nicks targeted to the CD44 gene in U2OS cells ectopically expressing RPA1-WT or its mutant derivatives and provided with a ssDNA donor for a-HDR by sequence analysis. **(D)** Maps of the fractional decrease in the number of base calls at each position within the indicated region spanning the gRNA 4 cleavage site in U2OS cells in cells treated as indicated and lacking or provided with a ssDNA donor complementary to the nicked strand (cN) to support a-HDR. **(E)** Maps of positions and spectra of SNVs within the indicated 65 bp region spanning nicks targeted by gRNA 4 to the CD44 gene in U2OS cells treated as indicated. The colored bar represents SNVs at each reference position: A, gray; C, orange; G, blue; T, red.

To identify factors that might promote resection at nicks, we first used the Traffic Light (TL) reporter [13] to determine the effect of depletion of candidate nucleases on frequencies of a-HDR at nicks in cells provided with a single-stranded oligonucleotide donor and co-depleted for BRCA2. Frequencies of canonical HDR (c-HDR) at DSBs in cells provided with a dsDNA plasmid donor were quantified in parallel. Frequencies of a-HDR at nicks were reduced in response to depletion of DNA2, but unaffected by depletion of EXO1 or MRE11; while frequencies of c-HDR at DSBs targeted to the same site were reduced in response to depletion of MRE11, DNA2, and to a lesser extent EXO1 (**S3A Fig**). A similar 2-fold reduction in a-HDR frequencies in response to depletion of DNA2 was evident at nicks targeted to either the transcribed or non-transcribed strand of the reporter construct, in a-HDR supported by ssDNA donors complementary to either the nicked (cN) or intact (cI) strand (**S3B Fig**). DNA2 possesses nuclease and motor activities that enable it to expose and resect DNA with either 5’-3’ or 3’-5’ polarity in DNA replication, recombination and DSB repair [8–12]. DNA2 is thus a plausible candidate to carry out resection that initiates repair at nicks resulting in an asymmetric distribution of mutations around the nick site (**Figs 1C, 1D**, **2D**, and **2E**), and to create a gapped substrate that can anneal with ssDNA donor complementary to the intact strand of the target in a-HDR pathways (**S3C Fig**).

DNA2 interacts with the trimeric factor RPA, which coats single-stranded gaps and free DNA ends exposed by DNA2 to prevent annealing and structure formation and coordinate end resection [14–16]. A human RPA trimer protects a single-stranded region approximately 20–30 nt in length [17,18]. RPA regulates the 5’ and 3’ resection activities of DNA2 [8,19], and interaction of DNA2/RPA with BLM or WRN helicase drives 5’ resection at DSBs in human cells [20,21]. To further understand resection at nicks, we therefore asked whether RPA participates in this process. This could not be studied by depletion analysis, because treatment with siRPA1, which targets the largest subunit of RPA, resulted in cell cycle arrest (**S4A Fig**). We therefore pursued an alternative strategy. The *S*. *cerevisiae* mutant Rfa1-t11 (K45E) supports normal replication but causes a defect in both mitotic and meiotic recombination [22–24]. Human RPA1 derivatives with mutations mapping near *S*. *cerevisiae* K45E were previously shown to support replication but were not analyzed for function in recombination [25,26]. By alignment we identified human RPA1 residues R41 or R43 as likely counterparts of *S*. *cerevisiae* K45 (**Fig 2A**). Based on this, we generated human RPA1 expression clones bearing the R41E and R43E single mutations as well as an R41/43E double mutation, and tested their effects on replication and HDR. Ectopic expression of RPA1-R41E, RPA1-R43E, or RPA1-R41/43E did not affect cell cycle (**S4B Fig**), but inhibited both a-HDR at nicks and c-HDR at DSBs (**Fig 2B**). Expression of either RPA1-R43E or RPA1-R41/43E reduced both a-HDR and c-HDR more than 5-fold. Note that inhibition was evident in cells expressing endogenous RPA, so these mutants exert a dominant negative effect on recombination. The RPA1-R43E single mutant was used for further analysis.

The roles of DNA2 and RPA in mutagenesis and a-HDR were then analyzed by amplicon sequencing. Sequence analysis showed that depletion of BRCA2 greatly stimulated a-HDR frequencies, which were reduced by co-depletion of DNA2 or by ectopic expression of RPA1-R43E (6.2- and 3.2-fold, respectively; **Fig 2C** left). In BRCA2-depleted cells, deletion frequencies were high in the absence of an HDR donor and reduced by co-depletion of DNA2 or by ectopic expression of RPA1-R43E (2.6- and 2.0-fold, respectively); and provision of an HDR donor reduced deletion frequencies more than 20-fold (**Fig 2C** right). Sequence analysis showed that either DNA2 depletion or ectopic expression of RPA1-R43E reduced deletions (**Fig 2D**) and SNVs (**Fig 2E**) on both sides of the nick site. These results support the view that availability of nicks as substrates for HDR or mutagenesis depends upon DNA2 and RPA, and further suggest that pathways of mutagenesis and a-HDR compete for the same DNA substrate.

### Cell cycle regulates DNA2/RPA-mediated resection at nicks and DSBs

**Fig 3:**
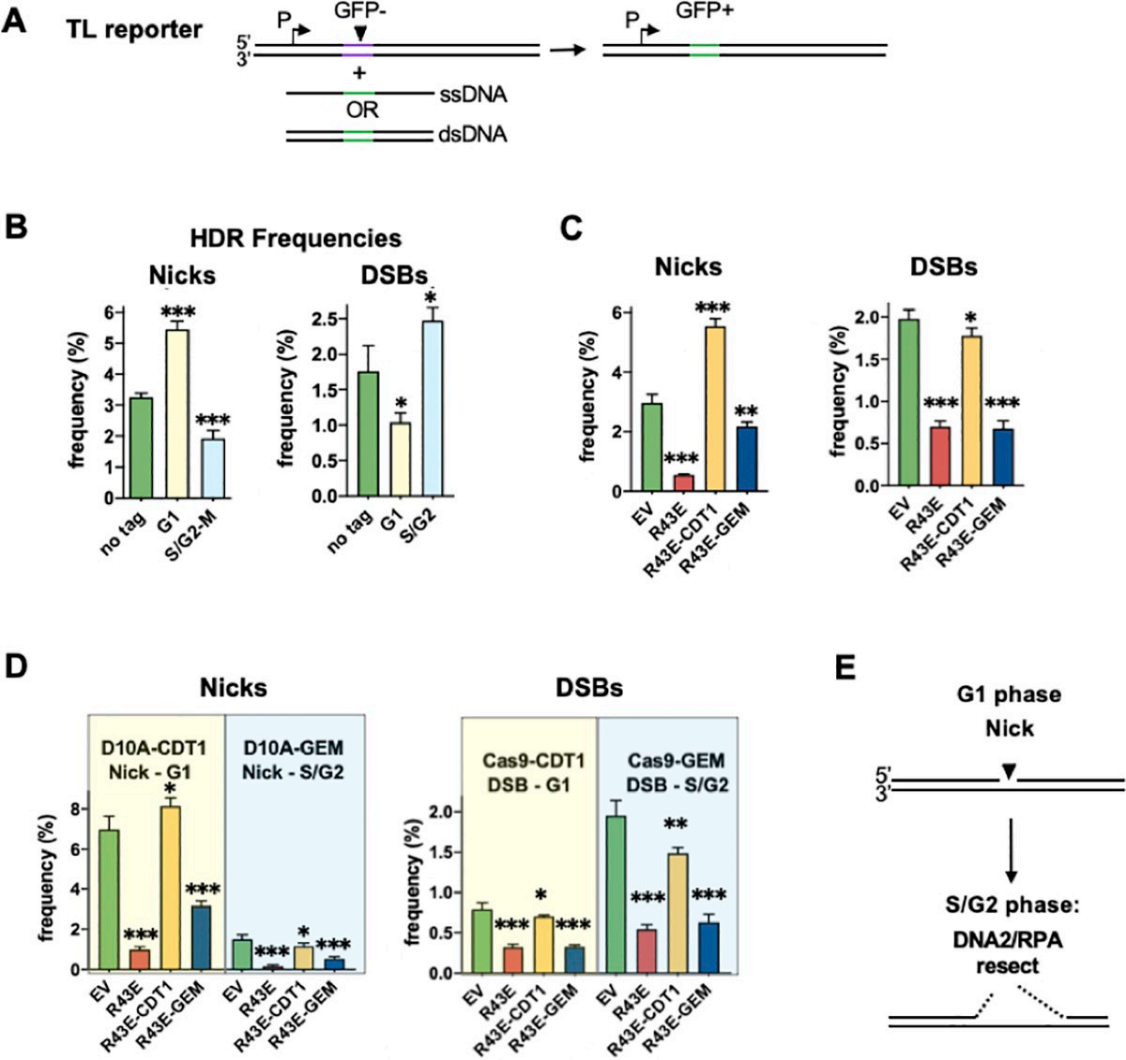
Cell cycle regulates HDR at nicks. **(A)** Traffic Light (TL) reporter [13] assay for HDR. The reporter carries a GFP gene rendered defective by a 38 bp insertion (purple), and driven by an upstream SFFV promoter (P). Repair is initiated by nicks or DSBs targeted to the insertion (arrowhead), and supported by either single-stranded DNA complementary to either strand or by a duplex DNA donor. HDR that replaces the 38 bp insertion with a 17 bp correction cassette (green) will correct the defective GFP gene, resulting in GFP^+^ cells. **(B)** Effect on HDR frequencies of restriction of nicks or DSBs to G1 or S/G2 phase. HDR frequencies were determined using the TL reporter in 293T TL cells transfected with untagged Cas9D10A or Cas9, or with derivatives bearing CDT1 or GEM tags to restrict cleavage to G1 or S/G2 phase, respectively. Cleavage was targeted by gRNA9 and supported by a cN ssDNA donor for a-HDR (nicks) or a plasmid donor for c-HDR (DSBs). Frequency values represent the mean ± SEM from a representative experiment; *, **, and *** indicate p<0.05, p<0.01, and p<0.001, respectively, for the frequency difference between indicated sample and sample transfected with untagged construct. **(C)** Effect on HDR frequencies at nicks and DSBs of restriction of the inhibitory activity of RPA1-R43E to G1 or S/G2 phase. Frequency values represent the mean ± SEM from a representative experiment; *, **, and *** indicate p<0.05, p<0.01, and p<0.001, respectively, for the frequency difference between indicated sample and sample transfected with empty vector (EV). **(D)** Effect on HDR frequencies at nicks and DSBs of cell cycle restriction of both cleavage and RPA1-R43E activities. Results obtained when cleavage was restricted to G1 or S/G2 distinguished by yellow or blue shading. Other details as in panel B. **(E)** Diagram of how a nick generated in G1 phase may persist to undergo resection later in cell cycle. Resection occurs within a zone extending 3’ and 5’ of the nick, consistent with participation of DNA2/RPA. While the analysis reported here does not directly connect the importance of RPA activity in S/G2 phase to its well-studied role in DNA2/RPA-mediated resection, it is consistent with that function.

DNA2 and RPA are regulated by cell cycle and most active in S phase [12]. c-HDR at DSBs, which depends on both these factors, is most efficient in S phase [27,28], and the dependence of a-HDR at nicks on these factors (**Fig 2**) suggested that a-HDR at nicks might also be most efficient in S phase. To test this, we assayed a-HDR and c-HDR in cells in which the nuclear activities of Cas9D10A, Cas9 or RPA were restricted to G1 or S/G2 phase of cell cycle. Expression constructs were generated bearing these factors fused to degrons derived from the CDT1 or geminin (GEM) cell cycle regulators [29], and the predicted restriction to G1 or G2/S phase, respectively, was confirmed by flow cytometry (**S5 Fig**). Relative efficiencies of HDR initiated during G1 or S phase were then quantified using the TL reporter assay (**Fig 3A**). Analysis of the effect of degron-tagged derivatives of Cas9D10A or Cas9 showed that, as previously reported [27,28], S/G2 phase DSBs initiated c-HDR more efficiently than G1 phase DSBs (generated by Cas9-GEM and Cas9-CDT1, respectively; **Fig 3B** right). In contrast, G1 phase nicks initiated a-HDR more efficiently than S/G2 phase nicks (generated by Cas9D10A-CDT1 and Cas9D10A-GEM, respectively; **Fig 3B** left).

Cell cycle dependence on RPA function was then assessed by determining HDR frequencies in cells in which the dominant negative RPA1-R43E mutant was expressed fused to cell cycle-regulated degrons. RPA1-R43E-CDT1 will inhibit HDR in G1 phase, and RPA1-R43E-GEM will inhibit HDR in S/G2 phase, so if HDR occurs preferentially in S/G2 phase, HDR frequencies are predicted to be reduced in cells expressing RPA1-R43E-GEM relative to control cells. This pattern was evident at both nicks and DSBs (**Fig 3C**). Thus, RPA promotes HDR at both nicks and DSBs more effectively in S/G2 phase than in G1 phase.

At nicks, a-HDR frequencies were unexpectedly higher in cells expressing RPA1-R43E-CDT1 than in control cells, an effect not evident at DSBs (**Fig 3C**). A possible explanation is that nicks generated in G1 phase undergo HDR most efficiently if they persist into S/G2 phase for repair. To address this possibility, HDR was assayed at nicks and DSBs initiated in either G1 or S/G2 phase, by Cas9D10A or Cas9 bearing CDT1 or GEM degron tags, in cells expressing dominant negative RPA1-R43E or its derivatives bearing CDT1 or GEM degron tags (**Fig 3D**). In all cases, HDR frequencies were higher in cells expressing RPA1-R43E-CDT1, which inhibits HDR in G1 phase, than in cells expressing RPA1-R43E-GEM, which inhibits HDR in S/G2 phase. Thus G1 phase nicks may persist to undergo repair later in cell cycle.

### DNA2 inhibits 1 bp deletions and insertions, but promotes longer deletions

**Fig 4:**
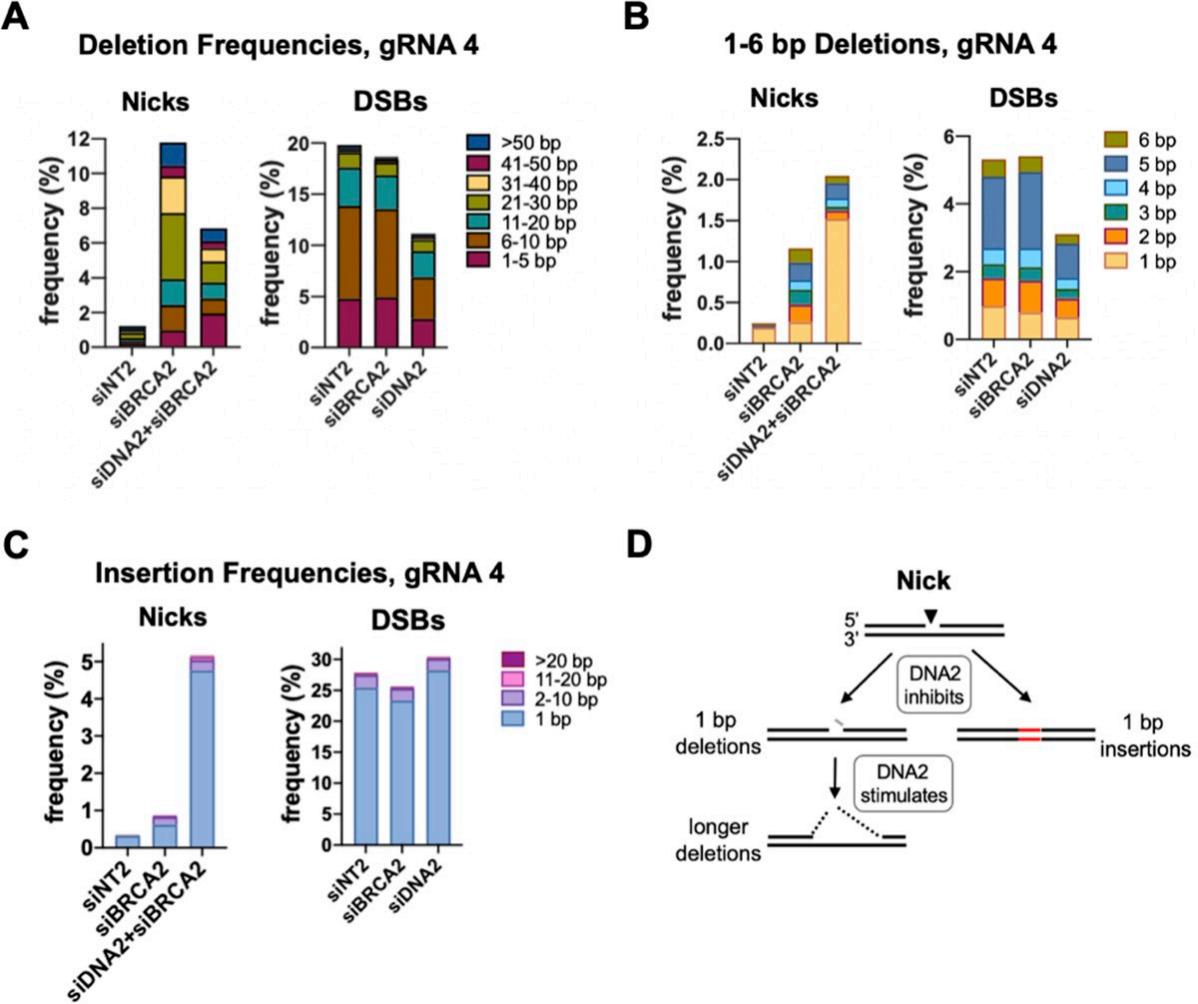
DNA2 inhibits 1 bp indels but promotes longer deletions at nicks targeted by gRNA 4. **(A)** Effects of depletion of BRCA2 and/or DNA2 on frequencies of deletions of indicated lengths at nicks or DSBs targeted by gRNA 4 to the CD44 gene in U2OS cells, determined by amplicon sequencing. **(B)** Effects of depletion of BRCA2 and/or DNA2 on frequencies of 1–6 bp deletions at nicks or DSBs targeted by gRNA 4 to the CD44 gene in U2OS cells. **(C)** Effects of depletion of BRCA2 and/or DNA2 on frequencies of insertions of indicated lengths at nicks or DSBs targeted by gRNA 4 to the CD44 gene in U2OS cells. **(D)** Diagram of regulation of deletions and insertions by DNA2. Inhibition by REV3 and DNA2 indicated by lines, with relative lengths reflecting relative magnitudes of inhibition. Resected DNA is indicated by dashes. Replicating DNA indicated by dashed black arrows; insertion by red font.

Amplicon sequencing was used to examine the frequencies and lengths of deletions and insertions at nicks and DSBs targeted by gRNA 4 in cells depleted for BRCA2 and DNA2 (**Fig 4**). In control cells (siNT2), the frequency of deletions was 20-fold lower at nicks than at DSBs (**Fig 4A**). At nicks, depletion of BRCA2 stimulated deletion frequencies more than 10-fold, and most deletions were >20 bp in length; while at DSBs, depletion of BRCA2 had little effect on deletion frequencies, and most deletions were <10 bp. At nicks, co-depletion of DNA2+BRCA2 reduced the overall deletion frequency nearly 2-fold relative to depletion of BRCA2 alone, reflecting reduced frequencies of longer deletions (**Fig 4A** left). At DSBs, the overall deletion frequency was also reduced nearly 2-fold by depletion of DNA2, but due primarily to a reduction in short deletions (**Fig 4A** right). Separate analysis of deletions 1–6 bp in length revealed that, at nicks, co-depletion of DNA2+BRCA2 caused a 6-fold increase in the frequency of 1 bp deletions but reduced the frequency of deletions of 2–6 bp; while at DSBs, DNA2 depletion reduced the frequencies of deletions of each length (**Fig 4B**).

Insertions were rare (<1%) at nicks in control cells (siNT2), and far more frequent at DSBs (**Fig 4C**). At nicks, insertion frequencies increased 3-fold in response to depletion of BRCA2 alone, and 17-fold in response to co-depletion of DNA2+BRCA2, reaching 5%; while at DSBs, depletion of BRCA2 or DNA2 had little effect on insertion frequencies. Surprisingly, the great majority of insertions at both nicks and DSBs were 1 bp in length, in all conditions tested.

Taken together, the results above identify an unexpectedly complex role for DNA2 at nicks targeted by gRNA 4 (**Fig 4D**). DNA2 promoted deletions at nicks, as it does at DSBs; however, at nicks this effect was restricted to deletions longer than 1 bp, and DNA2 specifically inhibited 1 bp deletions. DNA2 also inhibited the 1 bp insertions that constituted the majority of insertion events at nicks. At DSBs, 1 bp insertions frequently result from fill-in reactions at 5’ overhangs generated as a result of staggered cleavage by Cas9 [30–32]. However, there are no overhangs to fill in at a nick, so some other mechanism must generate these 1 bp insertions.

### REV7 and POLQ promote deletions longer than 1 bp at nicks

To identify pathways that contribute to mutagenic repair, amplicon sequencing was used to analyze libraries of sequences at nicks targeted by gRNA 4 to the CD44 gene in U2OS cells depleted for four factors—REV1, REV3, REV7 or POLQ—along with BRCA2. The activities of these factors have previously been extensively characterized. REV1 is a Y family polymerase that functions as a dCMP transferase, inserting a single C to bridge unusual structures during translesion synthesis [33]. REV3 and REV7 (also known as MAD2L2) are two components of the four-subunit B-family DNA polymerase POLζ (REV3/REV7/POLD2/POLD3), and they are recruited by REV1 and exchange with it to extend a distorted DNA primer-template [34]. REV7 also functions independently to protect telomeres and DSBs from resection, and to regulate NHEJ at DSBs as a component of the shieldin complex [35–41]. POLQ encodes POLθ, a DNA helicase and translesion polymerase that promotes repair of DSBs formed by a variety of mechanisms, including replication fork stalling, targeted DNA cleavage and ionizing radiation, by binding to and extending annealed short (2–6 bp) duplex regions; maintains genomic stability by preventing interhomolog recombination, which can result in loss-of-heterozygosity; and, conversely, promotes insertions and random chromosomal integration [42–48]. Libraries were generated in parallel from cell populations depleted first and then aliquoted for transfection with Cas9D10A or Cas9 RNPs, to provide a physiological control for depletion comparison with previously described activities at DSBs.

**Fig 5:**
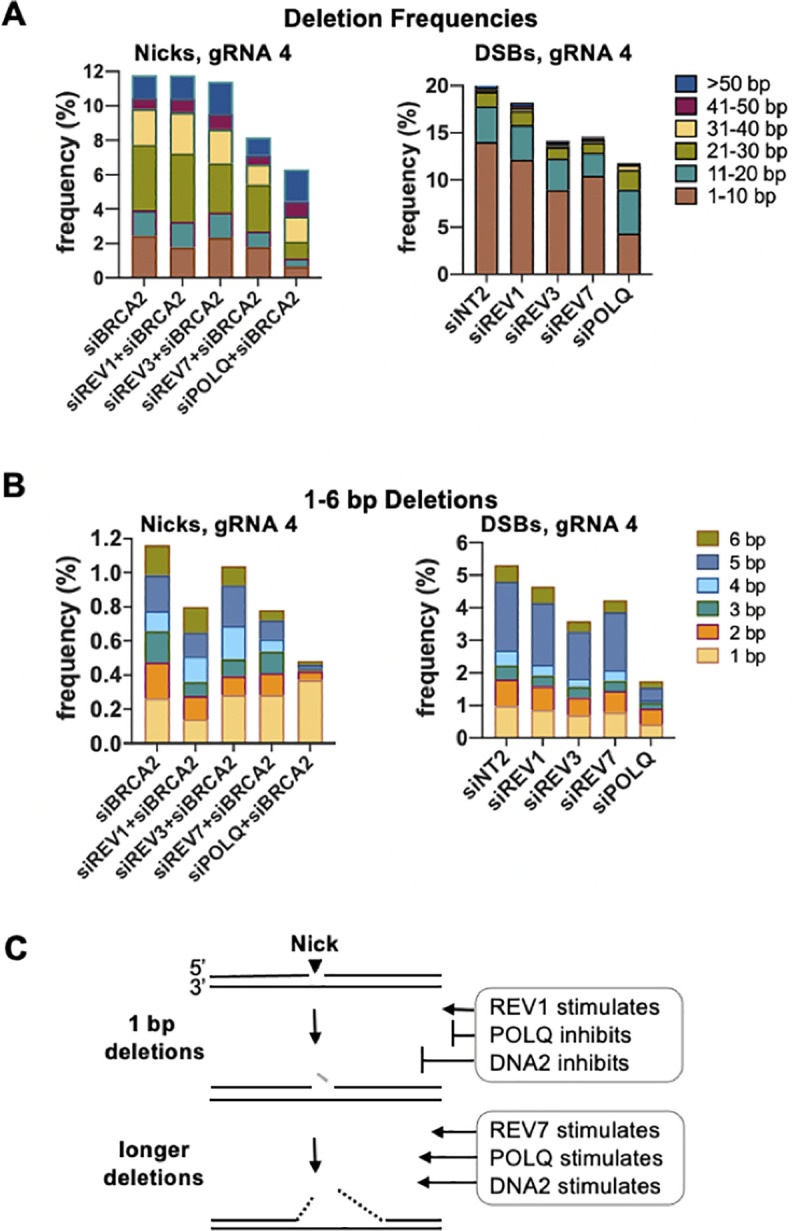
REV7 and POLQ promote deletions at nicks. **(A)** Effects of depletion of indicated factors on frequencies of deletions, binned by tens, at nicks and DSBs targeted by gRNA 4 to the CD44 gene of U2Os cells. **(B)** Effects of depletion of indicated factors on frequencies of 1–6 bp deletions. **(C)** Diagram of distinct regulation of 1 bp and longer deletions by REV1, REV7, POLQ and DNA2. Stimulation and inhibition indicated by lines, with relative lengths reflecting relative magnitudes of stimulation or inhibition.

Analysis of deletion frequencies showed that there was no effect of co-depletion of REV1 or REV3 and BRCA2 relative to depletion of BRCA2 alone, while co-depletion of REV7 or POLQ and BRCA2 reduced deletion frequencies (31% and 47%, respectively; **Fig 5A** left). At DSBs at the same site, reductions in deletion frequencies in the range of 10%-40% resulted from depletion of REV1, REV3, REV7 or POLQ (**Fig 5A** right).

Separate examination of short deletions (1–6 bp, **Fig 5B**) showed that at nicks, co-depletion of BRCA2 and REV1, REV7 or POLQ reduced the overall frequencies of deletions in this range, but with contrasting effects on 1 bp deletions, which were reduced 2-fold by REV1 depletion, unaffected by REV7 depletion, and increased nearly 2-fold by POLQ depletion. Depletion of these same factors, most notably POLQ, also reduced the frequencies of 1–6 bp deletions at DSBs. These results and the analysis of the effects of co-depletion of BRCA2 and DNA2 at nicks (**Fig 4B** and **4D**) both suggest that 1 bp deletions arise or are processed differently from longer deletions at nicks: 1 bp deletions are stimulated by REV1 and inhibited by POLQ and especially DNA2, and longer deletions are stimulated by REV7 and especially POLQ and DNA2 (**Fig 5C**).

### 1 bp insertions depend upon sequence context

**Fig 6:**
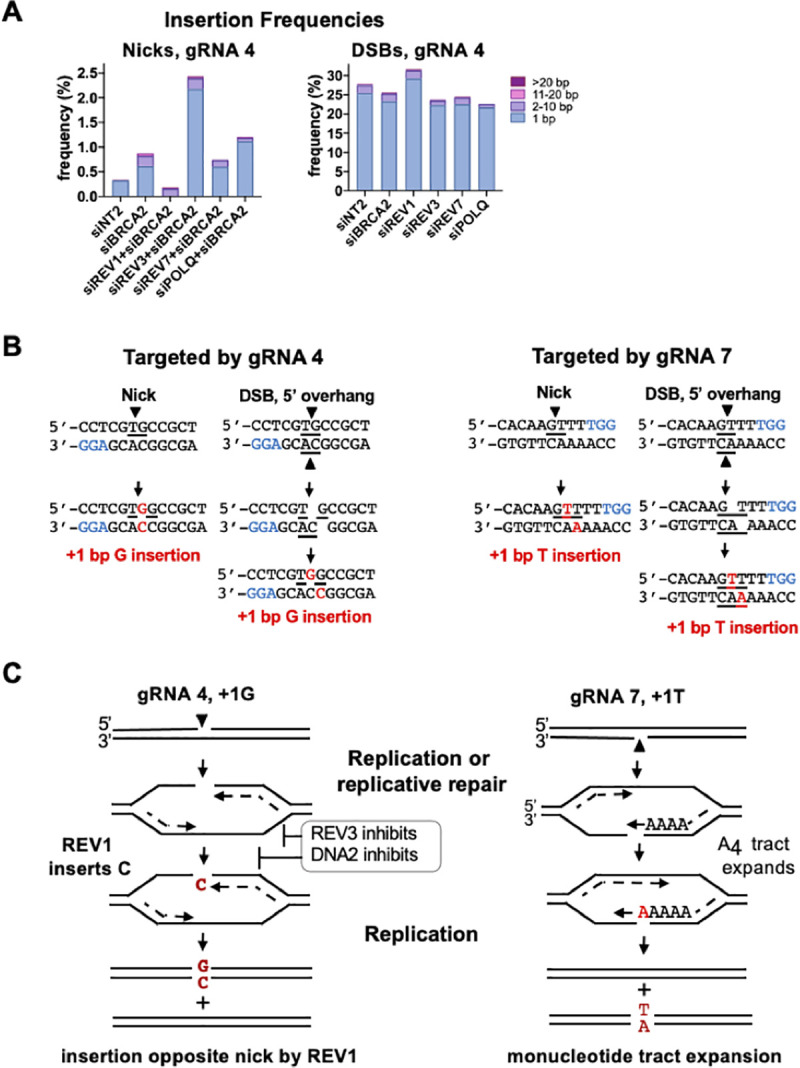
1 bp insertions at nicks targeted by gRNAs 4 and 7. **(A)** Effects of depletion of indicated factors on frequencies of insertions of indicated lengths at nicks and DSBs targeted by gRNA 4 to the CD44 gene in U2OS cells, as determined by amplicon sequencing. **(B)** Above, the 12 bp region surrounding the gRNA 4 (left) or gRNA 7 (right) target sites at nicks or DSBs (underlined, and strand or strands predicted to undergo cleavage indicated by arrowheads); PAM, blue font. Below, sequence changes due to 1 bp insertions at nicks or DSBs bearing 5’ overhangs, red font. For DSBs, the intermediate carrying 5’ overhangs is shown for clarity. These and other sequences shown illustrate the top DNA strand, which is the strand targeted for nicks by gRNA 4/Cas9D10A (**S1 Fig**). **(C)** Left, diagram of how REV1 may insert C opposite a nick targeted by gRNA 4 during replicative repair or replication, which templates addition of G on the opposite strand. Right, diagram of how replication slippage may expand the mononucleotide tract adjacent to the nick targeted by gRNA 7 from 4 to 5 nt. Following replication or mismatch repair, these will be scored as +1C and +1T insertions at the gRNA 4 and 7 sites, respectively.

Frequencies of insertions were nearly two orders of magnitude lower at nicks than at DSBs targeted by gRNA 4 (**Fig 6A** left). Depletion of BRCA2 increased insertion frequencies at nicks several-fold (**Fig 6A** left); and co-depletion of DNA2+BRCA2 increased the overall insertion frequency and the frequency of 1 bp insertions (**Figs 4C and 6C**). Co-depletion of REV1+BRCA2 caused a 3-fold reduction in the frequency of all insertions relative to BRCA2-depleted cells (0.29% vs 0.87% respectively) and eliminated 1 bp insertions. This immediately implicated REV1 as the source of 1 bp insertions at nicks targeted by gRNA 4. Insertion frequencies increased nearly 3-fold in response to depletion of REV3+BRCA2 relative to depletion of BRCA2 alone (2.44% vs 0.87%, respectively), suggesting that REV3 inhibits these 1 bp insertions. Sequence analysis further identified unexpected roles for REV3 and DNA2 in suppressing 1 bp insertions. Co-depletion of REV7+BRCA2 had little effect on insertion frequencies; and co-depletion of POLQ+BRCA2 caused a modest increase in the frequency of 1 bp insertions, and a modest reduction in longer insertions.

Frequencies of insertions at DSBs targeted by gRNA 4 were relatively unaffected by depletion of BRCA2 and modestly increased by depletion of REV1 (**Fig 6A** right). At DSBs, depletion of either REV3 or REV7 caused a reduction in insertion frequencies of similar magnitude (15%), which may reflect function of these factors in concert as components of POLζ. This contrasts with the increase in insertion frequencies at nicks in REV3- but not REV7-depleted cells (**Fig 6A** left), and suggests that REV3 and REV7 may function independently at nicks but not DSBs targeted by gRNA 4. At DSBs, depletion of POLQ caused a modest increase in 1 bp insertions and a decrease in longer insertions.

At nicks targeted by gRNA 4, insertion of a single G (+1G) on the nicked strand accounted for almost all insertion events in control (siNT2) cells and in other samples tested, except cells depleted for REV1 (**Figs S6A, 6B and 6C**). These +1G insertions could be mapped to the site of the nick (**Figs 6B** left and **S6D**). However, we note that this mapping is not definitive as we cannot formally distinguish between insertion of G at the nick site (to generate TGG, nick site underlined) or 1 bp 3’ (downstream) of it (to generate TGG). The +1 insertions frequently evident among repair products of DSBs generated by Cas9 result from filling-in of cleavage products that carry 1 bp 5’ overhangs rather than blunt ends [30–32]. Consistent with this, +1G insertions comprised the vast majority of insertions at DSBs targeted by gRNA 4 (**Figs 6B** left, **S7A, and S7B**).

Sequence analysis was also carried out on repair products of nicks and DSBs targeted by gRNA 7 to a site located 35 bp upstream of the gRNA 4 site in CD44 exon 1, and on the opposite (transcribed) DNA strand (**S1 Fig**). The frequency of insertions was comparable at nicks and DSBs targeted by either gRNA 4 or 7 (0.6–0.8% at nicks in cells depleted for BRCA2; 20% at DSBs**; Figs S6A and S7**). At nicks targeted by gRNA 7, insertion frequencies were not increased in response to co-depletion of DNA2 or REV3 with BRCA2, and only modestly reduced in response to co-depletion of REV1 and BRCA2; and the vast majority of insertions were +1T **(S7C Fig**). We cannot distinguish between +1T insertions that occurred at the nick site and at other positions in the mononucleotide run. Sequence analysis further showed that repair of DSBs targeted by gRNA 7 resulted in insertions of +1T on the top strand of the amplicon (**Figs 6B** right and **S7C**). This is consistent with the view that +1 insertions at DSBs result from filling-in 5’ overhangs of some cleavage products.

It is potentially significant that the sequences of +1 insertions at both nicks and DSBs targeted by gRNA 4 were identical, +1G; and that sequences of +1 insertions at both nicks and DSBs targeted by gRNA 7 were identical, +1T (**Fig 6B**). This could be coincidental. However, presuming that insertions at nicks occur at the nick site, the identity of insertions at nicks and DSBs can be explained mechanistically if the Cas9D10A nuclease occasionally cleaves not just one but both strands to generate DSBs bearing 1 nt 5’ overhangs rather than the predicted nicked substrates. If these aberrant cleavage products undergo repair by fill-in followed by ligation, sequencing will score them as 1 bp insertions.

Alternatively, the sequences of 1 bp insertions at nicks targeted by gRNAs 4 and 7 could reflect distinct mechanisms of repair active in either sequence context (**Fig 6C**). At nicks targeted by gRNA 4, the majority of insertions were +1G and the insertion frequency was considerably reduced (>10-fold) in response to depletion of REV1, a translesion polymerase with dCMP transferase activity. These +1C insertions could occur if REV1 bridged the discontinuity caused by the nick by adding a single C on the opposite strand, which would then template incorporation of a G on the opposite strand (**Fig 6C** left). In contrast, at the site targeted by gRNA 7, the length of a mononucleotide T-tract adjacent to the target site increased from 4 to 5 nt, raising the possibility that sequence expansion might occur as a result of slippage during replication (**Fig 6C** right).

### POLQ contributes to most SNVs at nicks and DSBs

**Fig 7:**
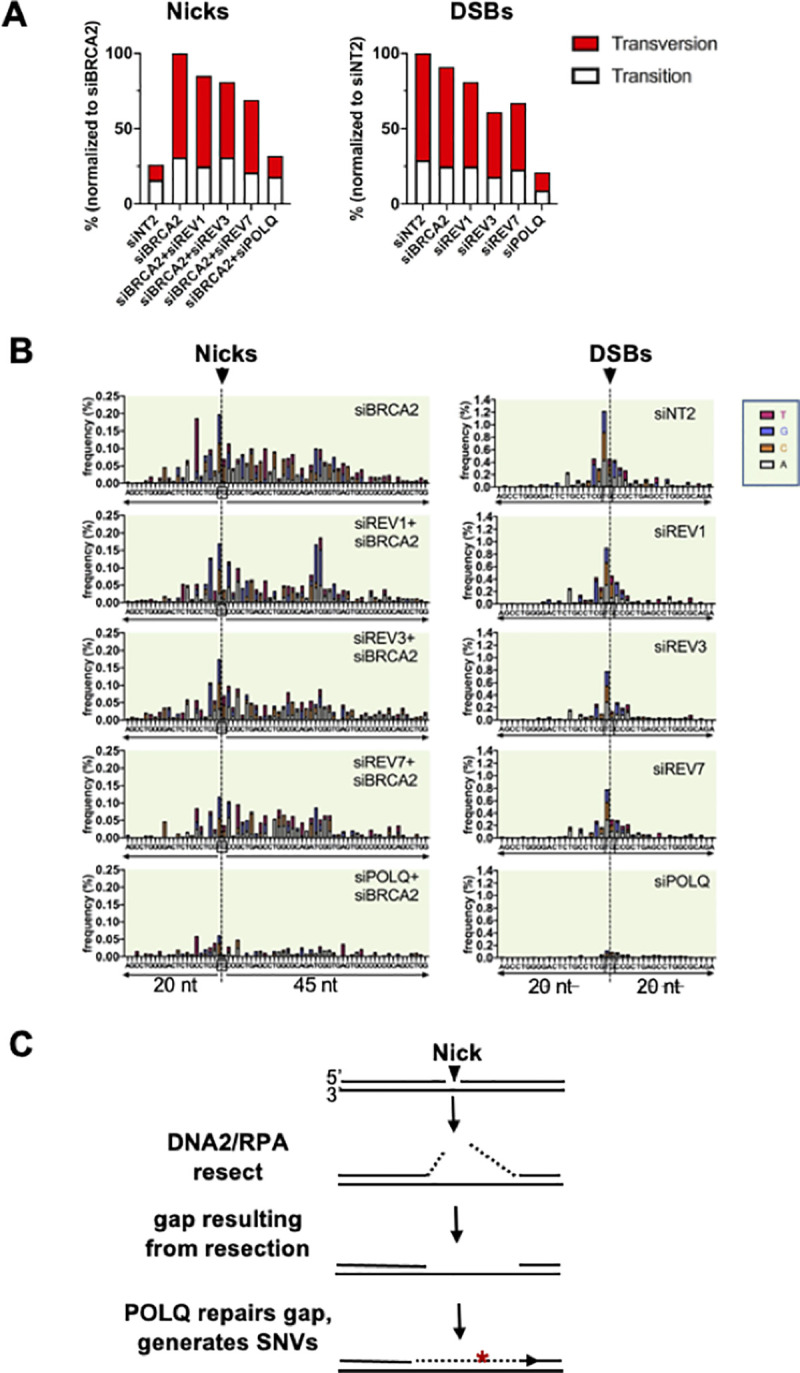
Single-nucleotide variants (SNVs). **(A)** Frequencies of SNVs at nicks and DSBs in the CD44 gene of U2OS cells depleted for indicated factors. To facilitate comparison of effects of different factors at nicks and DSBs, SNV frequencies were normalized to siBRCA2- or siNT2-treated cells, respectively, after subtraction of the background SNV frequency (0.26%) established by analysis of DNA from cells not targeted for cleavage by Cas9D10A. **(B)** Maps of positions and spectra of SNVs within the indicated 65 nt region spanning nicks or DSBs in cells depleted for indicated factors. The colored bar represents SNVs at each reference position: A, gray; C, orange; G, blue; T, red. **(C)** Diagram of mutagenic repair by POLQ of a gap generated by 5’ resection mediated by DNA2/RPA. Red asterisk indicates SNV; dashed lines, resected DNA.

SNV frequencies at nicks and DSBs were determined as described in Methods and normalized to siBRCA2- or siNT2-treated cells, respectively, to facilitate comparisons of the effects of each factor (**Fig 7A**). At nicks targeted by gRNA 4, frequencies of SNVs increased 4-fold in response to depletion of BRCA2, accompanied by a switch to a predominance of transversion mutations (from 25% to 70%). Co-depletion of REV1, REV3 or REV7 along with BRCA2 reduced SNV frequencies 15–30% relative to cells depleted of BRCA2 alone, but had relatively little effect on the fraction of transversion mutations. At DSBs, depletion of BRCA2 had little effect on SNV frequency or the fraction of transversions relative to control (siNT2) cells, and depletion of REV1, REV3 or REV7 reduced SNV frequencies with little effect on the fraction of transversions. Strikingly, SNV frequencies at both nicks and DSBs targeted by gRNA 4 were considerably reduced in response to depletion of POLQ. POLQ depletion also caused the fraction of transversions to diminish at nicks, though not at DSBs. These results identify an unexpected role for POLQ in generating SNVs at both DSBs and nicks.

Sequence analysis identified an asymmetric distribution of SNVs around the cut site at nicks but not at DSBs (**Fig 7B**). The pattern of distribution of SNVs at nicks was unaffected by co-depletion of REV1, REV3 or REV7 along with BRCA2 (**Fig 7B**); or by limiting the activity of DNA2 or RPA, although the latter two treatments did reduce SNV frequency (**Fig 2E**). Taken together, those observations suggest that resection by DNA2/RPA creates a gap that is filled in by POLQ to generate SNVs, as diagrammed in **Fig 7C**.

## Discussion

Nicks were long discounted as a source of genomic instability despite their frequent occurrence. The sequence analysis described here establishes the potential of nicks to promote deletions, insertions and SNVs, and identifies mutagenic signatures of DNA2/RPA, REV1, REV3, REV7 and POLQ at nicks. These results provide an outline for pathways of repair of DNA nicks mediated by factors familiar from DSB repair which in some cases function differently at nicks than at DSBs.

Nicks are normally protected from mutation by BRCA2, which limits the threat they pose to genomic instability. To permit sequence analysis to be carried out on a dataset that was not limited by a very low frequency of mutations, the pathways of nick repair were defined in cells depleted for BRCA2, which increased the frequencies of mutations and of a-HDR at nicks (**Figs 1** and **2**).

Availability of an HDR donor protected nicks from deletion. Protection may result from donor annealing that prevents access by DNA2 or other nuclease activities. The reduction in deletion frequencies caused by provision of a donor exceeded the increase in HDR frequencies, suggesting that donor annealing may protect a nick without necessarily leading to productive HDR.

At nicks, both deletions and SNVs were distributed asymmetrically, extending approximately 10 bp 5’ (upstream) and 25 bp 3’ (downstream) of the nick site, while no corresponding asymmetry was evident at DSBs (**Figs 1** and **2**). Asymmetry was evident at nicks targeted to either strand (**S2 Fig**), so it does not reflect the direction of transcription. Asymmetry may at least in part reflect resection by DNA2/RPA. DNA2 possesses both 3’ and 5’ exonucleolytic activities, and interaction with BLM or WRN helicase can drive its 5’ resection activity [8,19–21], consistent with predominately 5’ resection providing a source of asymmetry. The zone targeted for mutation coincides with the zone that forms an R-loop upon annealing to the 20 bp CRISPR gRNA, which may contribute to mutagenesis as R-loops are inherently unstable [49]. A single-stranded region approximately 20–30 nt in length is protected by a human RPA trimer [17,18], a dimension similar to that of the R-loop. Interaction of DNA2/RPA with single-stranded DNA within a limited region of this size at the nick site could explain the reduced frequencies of deletions and SNVs on both sides of the nick in cells in which the activity of DNA2 or RPA have been limited (**Fig 2**). RPA exhibits some sequence-specificity of DNA binding [17], which could contribute to distinct repair frequencies or outcomes at nicks targeted to different sites.

DNA2 and RPA are activated in S phase. Analysis of cell cycle dependence of repair leads to a model in which nicks generated in G1 phase may persist until S/G2 phase to undergo resection by DNA2/RPA (**Fig 3**). The evidence that depletion of DNA2 caused a 5-fold increase in 1 bp deletions at nicks targeted by gRNA 4 (**Fig 4B** and **4C**) raises the possibility that, in some contexts, binding of DNA2/RPA to the nick site confers physical protection. To carry this speculation a little further, cell cycle regulation of DNA2/RPA might even promote interaction with DNA in the absence of resection in G1 phase, then activate resection in S phase, providing a mechanism that enables nicks generated in G1 phase to persist into S phase for optimal HDR (**Fig 3**).

Sequence analysis showed that both REV7 and POLQ limit deletions at nicks targeted by gRNA 4 (**Fig 5**). REV7 is the non-catalytic subunit of the TLS polymerase POLζ (REV3/REV7), but depletion of REV3 did not affect the deletion frequency, making it unlikely that repair synthesis by POLζ limits deletions. The role of REV7 may instead be architectural, protecting nicks to enable their repair by other factors, as it does at DSBs. The evidence that DNA2 inhibited insertions at nicks while stimulating long deletions (**Fig 4**) raises the possibility that resection by DNA2/RPA generates a gap that becomes a source of deletions, and that POLQ may limit deletion by extending DNA 3’ ends at these gaps, as it does at DSBs [43,50]. In doing so, POLQ may create SNVs, which are largely POLQ-dependent at both nicks and DSBs (**Fig 7**). POLQ is highly error-prone, generating single base changes at a rate 10- to 100-fold higher than other family A polymerases on undamaged DNA templates in vitro [51]. POLQ is also mutagenic in vivo. It is one of several polymerases that contribute to immunoglobulin gene somatic hypermutation [52,53], where SNVs arise in the course of mutagenic repair of nicks caused by targeted deamination by AID [1]; and POLQ has also been shown to contribute to the mutation burden in solid tumors [54].

Insertions were analyzed at two different sites, targeted by gRNAs 4 and 7 to positions 35 bp apart and on opposite strands of the CD44 gene (**Figs 6, S6** and **S7**). The frequency of insertions was comparable at nicks and DSBs targeted by either gRNA (0.7% at nicks in BRCA2-depeleted cells; 18%, at DSBs), but the sequences inserted were different. Insertions were uniformly +1G at both nicks and DSBs targeted by gRNA 4, and at nicks and DSBs targeted by gRNA 7, they were uniformly +1T. This may be coincidental, but these results also raise the possibility that Cas9D10A "nickase" occasionally cleave both strands to generate DSBs with 5’ overhangs that are repaired by the same fill-in process active at cleavage products of Cas 9 that carry 5’ overhangs [30–32]. Alternatively, other mechanisms specific to sequence context could influence that pathways of +1 insertions (e.g. **Fig 6C**). Systematic characterization of repair products of nicks targeted to a number of different sites can distinguish between occasional generation of DSBs by Cas9D10A and more physiological repair mechanisms.

The results presented here demonstrate that nicks have considerable potential to contribute to mutagenesis. The increased frequencies of mutagenesis at nicks evident upon depletion of BRCA2 suggests that nick-initiated mutations will be especially frequent in tumors characterized by genetic or regulatory deficiencies that prevent RAD51 loading onto DNA ("BRCAness" [55]). About half of all solid tumors are currently treated with IR, including many BRCA-deficient tumors. IR generates far more nicks than DSBs, suggesting that the mutagenic signatures of these pathways outlined here will be enhanced in tumors treated with radiation.

## Materials and methods

### Culture and transfection of U2OS cells

U2OS cells were cultured at 37°C, 5% CO_2_ in DMEM (Gibco) containing 10% FBS, 2 mM L-glutamine, 100 units/ml penicillin and 100 μg/ml streptomycin (Gibco # 15140–122). For each siRNA transfection, 3x10^5^ U2OS cells were reverse transfected with 5 pmol of each siRNA (QIAGEN) using lipofectamine RNAiMAX (Thermo Fisher) in a 12-well tissue culture plate. At 24 hr post-transfection, cells were trypsinized, washed once with PBS, resuspended in 100 μl of Ingenio electroporation solution (Mirusbio), mixed with 10 pmol premade complex of CRISPR gRNA and Cas9 (DSBs) or Cas9D10A (nicks) recombinant protein (IDT), transferred to a cuvette and transfected with a 4D-Nucleofector (Lonza), using program CM-104. CRISPR gRNA 4 targeted exon 1 of the CD44 gene at the sequence 5’-cctCGTGGCCGCTGAGCCTGGCAC-3’ (PAM sequence is cct, shown in lowercase; cleavage targets the phosphodiester backbone between the underlined TG bases). Parallel transfection with an eGFP expression plasmid (240 ng; Invitrogen) was used to determine nucleofection frequencies (>90%). The frequency of indels was about 40% among molecules recovered from transfected control populations (siNT2) in which DSBs were targeted by gRNA 4/Cas9 (Figs 1C, 4A, and 4C). Taken together with the >90% transfection efficiency, this suggests that nearly half of all transfected cells were exposed to Cas9. Following transfection, cells were transferred to 6-well plates and further cultured for 3 days, at which time genomic DNA was isolated with the DNeasy Blood and Tissue Kit (QIAGEN).

For siRNA depletion, 3x10^5^ U2OS cells were incubated with 5 pmol siRNA using lipofectamine RNAiMAX (Thermo Fisher) in a 12-well tissue culture plate for 2 hr. Cells were the trypsinized, washed once with PBS, and resuspended in 100 μl of Ingenio electroporation solution (Mirusbio). After addition of 10 pmol premade complex of CRISPR gRNA and Cas9 or Cas9D10A recombinant protein (IDT) to each well, contents were transferred to a Lonza cuvette and transfection was carried out with the 4D-Nucleofector (Lonza), using program CM-104. Cells were then transferred to a 6-well plate for further culture and harvested at 72 hr post-transfection. Transfection frequencies were determined by parallel transfection with 240 ng of a control eGFP expression plasmid (IDT). Genomic DNA was isolated with the DNeasy Blood and Tissue Kit (QIAGEN) at 72 hr post-transfection. Parallel analysis of DSBs targeted to the same site provided a physiological control for depletion.

### siRNAs

siRNAs used were siNT2 (4390847), siBRCA2 (s2085), siEXO1 **(**s17502, S17503) and siMRE11 (s8959, s8960) from Thermo Fisher Scientific; and siDNA2 (SI05067622; SI00370475; SI04293989; SI04309977), siPOLQ (SI02665215, SI00090083, SI00090076, SI0009006); siREV1 (REV1L; SI03090171, SI00115311, SI00115304, SI00115297); siREV3 (SI00045647, SI00045626, SI03058643, SI03120278) and siREV7 (MAD2L2; SI00087710, SI00087703, SI00087696, SI00087689) from Qiagen.

### Amplicon sequencing

Genomic DNA for sequencing was isolated 6–7 days post-transfection and prepared as previously described [48]. Genomic DNA was isolated from populations of 10,000–30,000 transfected cells without sorting CD44- from CD44+ cells to avoid underestimating mutation frequency, as some mutations do not affect the CD44+ phenotype. To enable direct comparisons of mutation frequencies among samples, and to control for depletion, libraries were generated in parallel from identically depleted cells targeted with nicks or DSBs. Expression of CD44 does not affect transfection efficiency or proliferation in culture, so cells were not sorted prior to collection. Approximately 1x10^6^ cells were used for genomic DNA preparation. Sequencing libraries were prepared followed a modified version of Safe-seq [56] in which genomic DNA underwent limited amplification to add unique molecular identifiers (UMIs), and was then further amplified to generate material for NGS analysis on an Illumina platform. The average coverage of mapped, UMI collapsed and aligned reads ranged from 43,500X to 67,000X. The coverage was calculated as N*L/G, where the number of aligned reads (N) after making consensus sequences were ranged from 78,000 to 120,000; the average read length (L) was 268, and the amplicon size (G) is 480.

Primers containing UMIs (5’-TCGTCGGCAGCGTCAGATGTGTATAAGAGACAGNNNNNNNNNNACTTCGGTCCGCCATCCTCGTC-3’ and 5’-GTCTCGTGGGCTCGGAGATGTGTATAAGAGACAGNNNNNNNNNNGCAAATCCCAGCCCTGCTTTC-3’) were used to amplify a 323 bp region of exon 1 of the CD44 gene spanning the cut site for 4 cycles using Phusion Hot Start II DNA Polymerase (Thermo Fisher Scientific). PCR products were purified with Ampure XP beads (Beckman Coulter) and further amplified using a pair of primers containing Illumina Nextera P5 and P7 adapter sites for a total of 25 cycles. Primers for both amplification steps were obtained from IDT. PCR products (480 bp) were purified with 0.7 volume of Ampure XP beads (Beckman Coulter), and reversely cleaned up by 7% Polyethylene Glycol 8000 (PEG) in 875 mM NaCl solution (final concentration) followed by a 0.7 volume of Ampure XP beads purification. Purified PCR products were then pooled in equal molar ratio, and sequenced on Illumina MiSeq (600 cycles V3, paired ended reads) at the Fred Hutchinson Cancer Research Center.

Reads were first converted to unaligned SAM files using FastqToSam command from PICARD tools. Then the UMI sequences were extracted and converted to "ab/ba" format where ’a’ and ’b’ are the tag sequences from Read 1 and Read 2 using an inhouse script. Remaining reads were then assembled to BAM files in which reads having the same UMI were assembled to consensus sequences using single strand consensus sequence (SSCS) assembly in FASTQ format. Remaining reads were aligned with the reference sequence and analyzed using two established methods. (1) In an approach designed to accurately call SNVs by scoring only mutations found at complementary positions on both DNA strands, SSCS read 1 and read 2 were aligned by Burrows-Wheeler Aligner (BWA) MEM and realigned around INDELs by GATK. Variants were called using Samtools mpileup. INDELs were normalized using bcftools. Output of mutation frequencies, SNVs, insertions, deletions at each position was generated by a python program derived from [57]. (2) SSCS reads were analyzed using CRISPResso2 [58].

INDELs and SNVs from the resulting Variant Call Format (VCF) sequence data files (S1 Data) were then analyzed using Excel. Sequence analysis of uncut DNA enabled quantification of background frequencies which were subtracted from reported frequencies prior to displaying data using GraphPad Prism. Libraries from several independent experiments were sequenced and analyzed; representative data from a panel of libraries is presented here. Libraries analyzing the effects of depletion of these factors and of DNA2 were constructed concurrently, so frequencies of mutagenesis could be directly compared. Background frequencies for this representative panel of libraries were: insertions, 0.47%; deletions, 0.3%; SNVs, 0.26%. These frequencies were subtracted from results shown.

### HDR reporter assays

Cell culture, transfection, depletion and reporter assays using the Traffic Light (TL) construct in 293T TL cells were carried out as previously described in detail [59]. In brief, the CRISPR RNP was delivered using RNAiMAX according to the manufacture’s recommendation (IDT). To determine nucleofection or RNAiMAX transfection frequencies, Alt-R CRISPR-Cas9 tracrRNA- ATTO 550 (IDT) was used. Transfection frequencies were typically greater than 90%. Unless otherwise specified, DNA DSBs or nicks were targeted by gRNA9 to the sequence 5’-TAAAGCTAAGAGCTCACCTAcgg-3’ (PAM lowercase, target site between underlined bases). Donors for HDR were either the duplex plasmid pCS14GFP [3] or the 99 nt single-stranded oligonucleotide SSO-2, complementary to the strand nicked by gRNA9. The sequence of SSO-2 is shown below, where uppercase letters denote arms of homology between SSO-2 and the target, and lowercase letters indicate the central region of heterology that must replace sequence in the target to generate a functional GFP gene:

SSO-2: 5’-TGGACGGCGACGTAAACGGCCACAAGTTCAGCGTGTCCGGCgagggtgagggcgatgcCACCTACGGCAAGCTGACCCTGAAGTTCATCTGCACCACCG-3’

Frequencies of GFP+ cells (HDR) were determined three days post-transfection by flow cytometry, and normalized for transfection efficiency as determined by parallel transfection of a GFP expression plasmid. Data were collected using a BD Biosciences LSR II Flow Cytometer. Each set of assays was performed in triplicate, and a mean frequency of HDR was determined. The values presented represent the mean ± SEM from a representative experiment. Two-tailed T-tests were performed using Microsoft Excel (2015) to determine if the differences between HDR and mutEJ frequencies at different stages of cell cycle were statistically significant. Results are presented as frequencies of HDR among transfected cells, as measured in parallel by transfection with a GFP expression construct.

### Expression constructs

RPA1 expression constructs were generated as derivatives of a RPA1-WT expression construct pLX304-hRPA1 bearing an N-ter V5 tag (Addgene Plasmid #25890). The R41E, R43E, and R41/43E mutations were made by site-directed mutagenesis [60] using the following primers (mutation sites underlined): for RPA1-R41E: 5’-CCGCCGGAATATCGACTGCTCATGAGTGATGGATTGAACACTCTATCC-3’ and 5’-AGCAGTCGATATTCCGGCGGACTATTCCCCGTAGTAATGGGACGGATGTTG-3; for RPA1-R43E 5’-CCGCCGCGTTATGAACTGCTCATGAGTGATGGATTGAACACTCTATCC-3’ and for RPA1-R41/43E, 5’-AGCAGTTCATAACGCGGCGGACTATTCCCCGTAGTAATGGGACGGATGTTG-3’; 5’-CCGCCGGAATATGAACTGCTCATGAGTGATGGATTGAACACTCTATCC-3’ and 5’-AGCAGTTCATATTCCGGCGGACTATTCCCCGTAGTAATGGGACGGATGTTG-3’.

C-terminal CDT1 and GEM tags were added using a modified overlap extension PCR cloning method [61]. The CDT1 and GEM tags were amplified from pCDNA-Cas9-CDT1 using the primers 5’-GCCTATCCCTAACCCTCTCCTCGGTCTCGATTCTACGAGCGGTGGAGGCGGTTCACGCCAATTCGCCACCCCCAGCCCCG-3’ and 5’-CTTAACGCGCCACCGGTTAGCGCTAGCTCATTACTAGATGGTGTCCTGGTCCTGCGCGGATG-3’ to amplify the CDT1 tag from pCDNA-Cas9-CDT1; and from pCDNA-Cas9-GEM using the primers 5’-GCCTATCCCTAACCCTCTCCTCGGTCTCGATTCTACGAGCGGTGGAGGCGGTTCACGCCAATTCGCCACCATGAATCCCAGTATGAAGCAGAAAC-3’ and 5’-CTTAACGCGCCACCGGTTAGCGCTAGCTCATTACTACAGCGCCTTTCTCCGTTTTTCTGCC-3’. The amplified CDT1 and GEM tags were then integrated into pLX304-hRPA1 by PCR. Constructs were verified by both restriction digestion and sequencing; and cell cycle regulation of protein stability was verified by flow cytometry (**S2 Fig**).

To generate the pCDNA-Cas9-CDT1, pCDNA-Cas9-GEM, pCDNA-Cas9D10A-CDT1 and pCDNA-Cas9D10A-GEM expression constructs, we replaced the T2A-BFP tag in both pCDNA-Cas9-T2A-BFP and pcDNA-Cas9D10A-T2A-BFP [3] with the mKO2-hCDT1(30–120) and mAG-hGEM(1–110) cell cycle tags [29]. Cloning was carried out as follows: First, the MfeI site in both pCDNA-Cas9-T2A-BFP and pCDNA-Cas9D10A-T2A-BFP was destroyed by MfeI digestion, fill-in and religation. The plasmids were then digested with NotI and XbaI, to remove the T2A-BFP cassette, which was replaced with a short duplex, Linker MCS, which carries NotI-MfeI-HpaI-NheI sites, and a linker encoding a pentapeptide (Gly-Gly-Gly-Gly-Ser) between the NotI and MfeI sites. We previously used this approach to confer cell cycle restriction to nuclear stability of Activation-Induced Deaminase (AID) [62], and these tags are referred to previously and herein as the CDT1 and GEM tags. To generate CDT1-tagged constructs pCas9D10A-mKO2-CDT1, pCas9-mKO2-CDT1, and Cas9 and Cas9D10A expression plasmids (see above) were digested with MfeI and NheI, and an EcoRI/XbaI fragment bearing the CDT1 cassette from pCSII-EF-mKO2-hCDT1(30–120) [29] was inserted between those sites. To generate GEM-tagged constructs pCas9D10A-mAG-GEM and pCas9-mAG-GEM, the CDT1-tagged constructs were digested with NheI, partially filled in using only dCTP and dTTP, then digested with MfeI to remove the cassette bearing mKO2 and the CDT1 tag; and ligated to a cassette carrying the GEM tag, generated by digestion of pCSII-EF-mAGhGEM(1–110) [29] with HinDIII, partially filled in using only dGTP and dATP, then digested with EcoRI. To generate constructs pCas9D10A-CDT1, pCas9D10A-GEM, pCas9-CDT1 and pCas9-GEM, cassettes encoding the mAG and mKO2 fluorescent proteins were removed by digestion with NotI, overhangs filled to maintain reading frame, and plasmids religated. All constructs were verified by both restriction digestion and sequencing; and cell cycle regulation of protein stability was verified by flow cytometry (**S3 Fig**).

### Cell cycle analysis by flow cytometry

To analyze ectopic expression of CDT1- or GEM-tagged RPA1-R43E, 1x10^6^ U2OS or HEK293T cells were transfected with 750 ng of plasmid, and at 36 hr post-transfection cells were harvested by trypsinization (0.05%) and washed twice with cold PBS. Cells were then fixed and permeabilized by incubation with 500 μl of 1x Foxp3 /Transcription Factor Fixation/Permeabilization solution (Invitrogen) for 30 min at room temperature; washed twice with the same buffer; and resuspended in 500 μl of the same buffer. Samples were then divided into two 250 μl aliquots and stained at room temperature for 1 hr with anti-RPA1 antibodies (rabbit, Abcam ab79398; 1:100) to detect endogenous RPA1 or anti-V5 tag antibodies (mouse, BioRad; 1:100) to detect ectopically expressed RPA1. Cells were then washed once in 1 ml PBS and stained with DAPI (10 μg/ml, ThermoFisher) in 10 mM Tris, pH 7.4, 150 mM NaCl, 2 mM CaCl_2_, 22 mM MgCl_2_, 0.1% NP-40, 0.05 mg/ml BSA and 10% DMSO, and then resuspended in 200 μl PBS containing 1% FBS and analyzed by flow cytometry on a BD LSR II instrument, set to record 50,000 cells per sample. Data were analyzed using FlowJo software (version 9.6).

To analyze cell cycle dependence of expression of CDT1- or GEM-tagged Cas9D10A, 3x10^5^ U2OS or HEK293T cells were transfected with 200 ng of expression plasmid, and at 36 hr post-transfection cells were harvested by trypsinization (0.05%), washed twice with cold PBS, then stained with DAPI and further analyzed as above.

## Supporting information

S1 Fig Target sites of gRNAs 4 and 7 in exon 1 of the CD44 gene in U2OS cells. **(A)** Diagram of nicks targeted to the non-transcribed or transcribed strands by gRNAs 4 and 7, respectively. P, promoter; arrowheads, nick target sites. **(B)** Sequence of the region of the CD44 gene targeted by gRNAs 4 and 7. Target sites, red font; regions of DNA that form hybrids with the CRISPR gRNAs, underlined.
(PDF)

S2 Fig Asymmetry of mutations targeted by gRNAs 4 and 7. **(A)** Diagram of predominately 5’ asymmetric resection at nick sites on opposite DNA strands. **(B-D)** Maps of mutations (including deletions, SNVs and insertions) flanking nicks or DSBs targeted to sites 35 bp apart by gRNA 4 or gRNAs 4 and 7. Cells were depleted with siNT2 (control) or siBRCA2, as indicated. Each panel maps the 240 bp region centered on the gRNA 4 target site, with site(s) cleaved indicated by arrowhead. Frequencies of mutations at the gRNA 4 site shown at the top of the y-axis.
(PDF)

S3 Fig Treatment with siDNA2 inhibits both a-HDR and c-HDR. **(A)** Effects of DNA2, EXO1 or MRE11 depletion on frequencies of a-HDR or c-HDR at nicks or DSBs, respectively. Frequencies were normalized relative to frequencies in 293T TL cells treated with siBRCA2 (nicks) or siNT2 (DSBs). Cleavage was targeted by gRNA9 (see panel B) and supported by a cN ssDNA donor for a-HDR (nicks) or a plasmid donor for c-HDR (DSBs). **(B)** Left, diagram of the cleavage sites for gRNA 2 and gRNA 9 in the TL construct (arrowheads), with the promoter (P) upstream. Right, effects of depletion of DNA on frequencies of HDR at nicks or DSBs targeted to the TL reporter construct in 293T TL cells by the indicated gRNA. HDR was supported at nicks by a donor complementary to the nicked or intact strand (cN or cI, respectively), in cells treated with siNT2, siBRCA2 or siDNA2+siBRCA2, as indicated; or at DSBs by a dsDNA donor, in cells treated with siNT2 or siDNA2. Frequency values represent the mean ± SEM from a representative experiment; and * and *** indicate p<0.05 and p<0.001, respectively, for the frequency difference between indicated sample and sample treated with siBRCA2 (nicks) or siNT2 (DSBs). **(C)** Working model for the role of DNA2 resection in a-HDR at nicks supported by a cN or cI donor. Results in panel B show that DNA2 promotes a-HDR by both pathways, and the first step shown in resection 3’ of the nick. The cN or cI donors anneal as shown, and processing then generates a heteroduplex which is resolved by replication.
(PDF)

S4 Fig RPA1-R43E inhibits replication but not HDR. **(A)** Cell cycle profiles of U2OS control cells (siNT2) or cells treated with siDNA2 or siRPA1. **(B)** Cell cycle profiles of U2OS control cells (mock) or cells expressing RPA1-WT, RPA1-R41E, RPA1-R43E or the RPA1-R41/R43E double mutant.
(PDF)

S5 Fig CDT1 and GEM tags restrict protein expression to G1 or S phase. **(A)** Cell cycle profiles of 293T cells transfected with Cas9D10A-mKO2-CDT1 or Cas9D10A-mAGGEM expression constructs, showing either the entire population or the population gated for PE+ (mKO2) or GFP+ (mAG) cells. **(B)** Cell cycle profiles of cells mock-transfected or transfected with CDT1- or GEM-tagged derivatives of RPA1-R43E bearing a C-terminal V5 tag, and stained with anti-RPA antibody, which detects endogenous and ectopically expressed RPA; or with anti-V5 antibody, which detects ectopically expressed RPA1-R43E.
(PDF)

S6 Fig Single base insertions in the region spanning the gRNA 4 nick site. **(A)** Tabulated effects of depletion of indicated factors on frequencies of 1 bp insertions of A, C, G or T or all nucleotides at nicks targeted by gRNA 4. These and other sequences shown illustrate the top DNA strand, which is the strand targeted for nicks by gRNA 4/Cas9D10A (**S1 Fig**). **(B)** Graph of effects of depletion of indicated factors on frequency of molecules bearing +1bp insertions of A, C, G or T at the gRNA 4 target site. **(C)** Sequence of predominant 1 bp insertions at the gRNA 4 target site. Nick site, underlined; PAM, blue font; insertion, red font. **(D)** Tables show nucleotides identified at indicated positions in products of repair containing a 1 bp insertion within the 4 bp region spanning nick site, in U2OS cells treated as indicated Positions are numbered -2, -1, +1, +2, relative to the nick site in the reference sequence GT/GC, where the slash marks the site at which the nick targeted to the CD44 gene by gRNA 4 cleaves the phosphodiester backbone.
(PDF)

S7 Fig Single base insertions at nicks and DSBs at the gRNA 4 and 7 target sites. **(A)** Left, effects of depletion of indicated factors on frequencies of 1 bp insertions of A, C, G or T at DSBs targeted by gRNA 4. Right, the 12 bp region surrounding the gRNA 4 target site (underlined) at DSBs bearing 5’ overhangs; and +1G insertion at that site (red). PAM, blue font. The postulated repair intermediate is shown for clarity. **(B)** Left, effects of depletion of indicated factors on frequencies of 1 bp insertions of A, C, G or T at nicks targeted by gRNA 7. Right, the 12 bp region surrounding the gRNA 7 target site (underlined in nicked strand) and +1T insertion at that site (red). PAM, blue font. **(C)** Left, effects of depletion of indicated factors on frequencies of 1 bp insertions of A, C, G or T at nicks targeted by gRNA 7. Right, the 12 bp region surrounding the gRNA 7 target site (underlined) at DSBs bearing 5’ overhangs and +1T insertion at that site (red). PAM, blue font. The postulated repair intermediate is shown for clarity.
(PDF)

S1 Data Variant Call Format (VCF) sequence data. (ZIP)
