# Supplementary material for: Pathways and signatures of mutagenesis at targeted DNA nicks
Source: PLoS Genet. 2021 Apr 15;17(4):e1009329. doi: 10.1371/journal.pgen.1009329 (PMC8078790; doi:10.1371/journal.pgen.1009329)
Supplement: S1 Fig — (A) Diagram of nicks targeted to the non-transcribed or transcribed strands by gRNAs 4 and 7, respectively. P, promoter; arrowheads, nick target sites. (B) Sequence of the region of the CD44 gene targeted by gRNAs 4 and 7. Target sites, red font; regions of DNA that form hybrids with the CRISPR gRNAs, underlined. (PDF) [file pgen.1009329.s001.pdf]

**A**

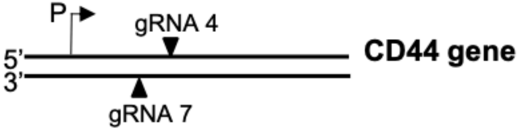


**B**

<u>**CD44 amplicon sequence**</u>

5'–TCGGTCCGCCATCCTCGTCCCGTCCTCCGCCGGCCCCTGCCCCGCGCCCAGGGATCCTCCAGCT

[gRNA 7]
CCTTTCGCCCGCGCCCTCCGTTCGCTC<u>CGGACACCATGGACAA**GT**TT</u>TGGTGGCACGCAGCCTGGGG

[gRNA 4]
ACTCTGCCT<u>CG**TG**CCGCTGAGCCTGGCGC</u>AGATCGGTGAGTGCCCGCCGCAGCCTGGGCAGCAAGAT

GGGTGCGGGGTGCTCAGCGCGGACCCGGCGGCAGCCCCTCCGGCTGAGTCGGCCCTGGGGGACTG–3'
