# Supplementary figures and images for: Pathways and signatures of mutagenesis at targeted DNA nicks
Source: PLoS Genet. 2021 Apr 15;17(4):e1009329. doi: 10.1371/journal.pgen.1009329 (PMC8078790; doi:10.1371/journal.pgen.1009329)

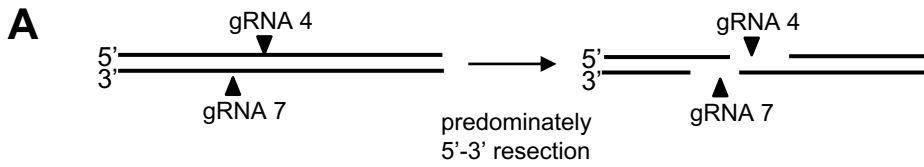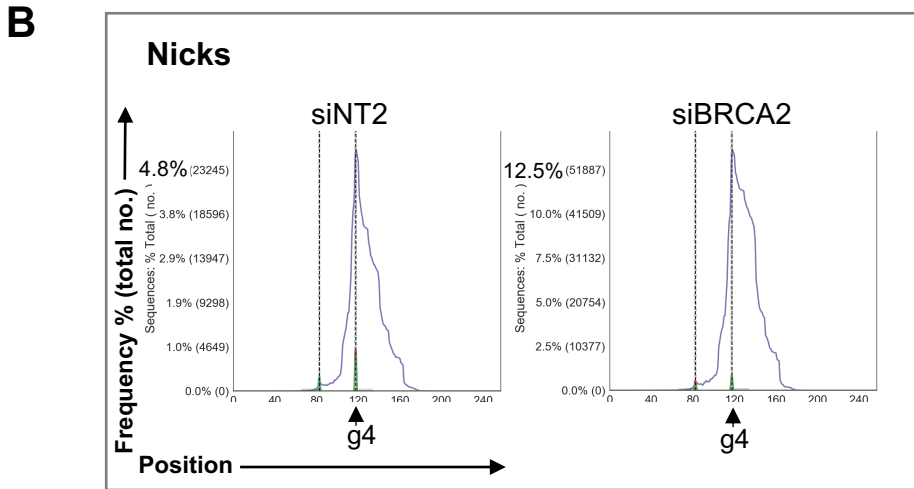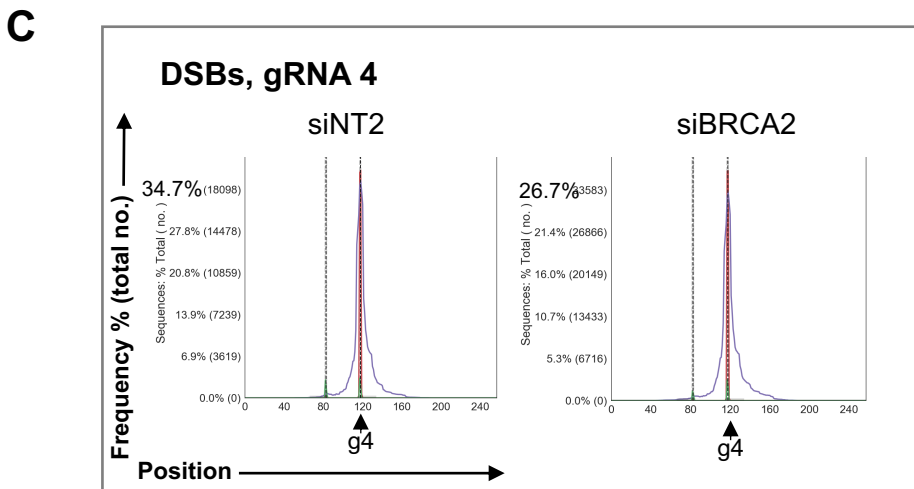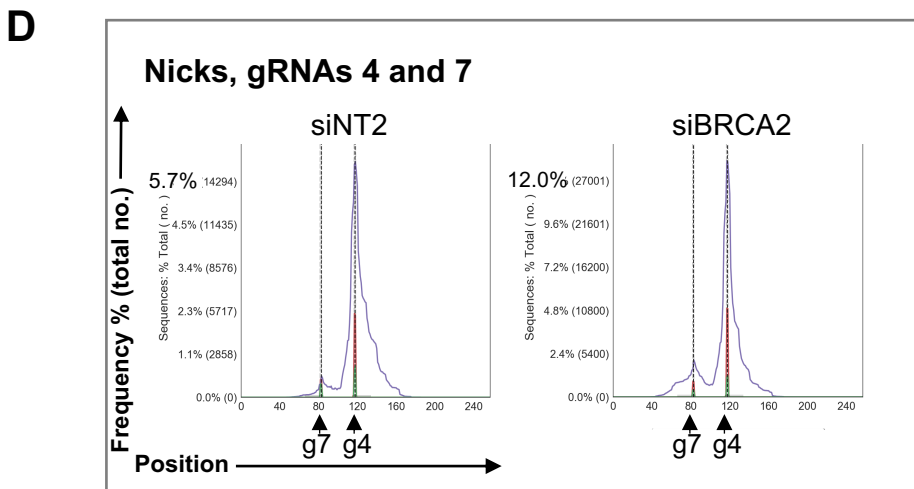

Supplement: S2 Fig — (A) Diagram of predominately 5’ asymmetric resection at nick sites on opposite DNA strands. (B-D) Maps of mutations (including deletions, SNVs and insertions) flanking nicks or DSBs targeted to sites 35 bp apart by gRNA 4 or gRNAs 4 and 7. Cells were depleted with siNT2 (control) or siBRCA2, as indicated. Each panel maps the 240 bp region centered on the gRNA 4 target site, with site(s) cleaved indicated by arrowhead. Frequencies of mutations at the gRNA 4 site shown at the top of the y-axis. (PDF) [file pgen.1009329.s002.pdf]

**A**

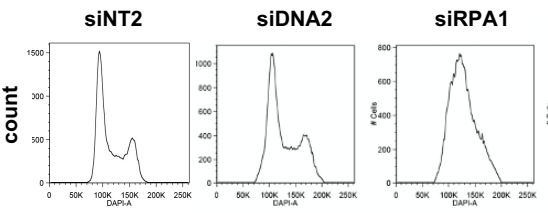

**B**

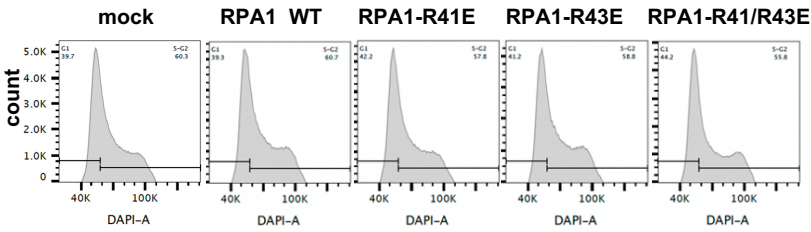

Supplement: S4 Fig — (A) Cell cycle profiles of U2OS control cells (siNT2) or cells treated with siDNA2 or siRPA1. (B) Cell cycle profiles of U2OS control cells (mock) or cells expressing RPA1-WT, RPA1-R41E, RPA1-R43E or the RPA1-R41/R43E double mutant. (PDF) [file pgen.1009329.s004.pdf]

**A**

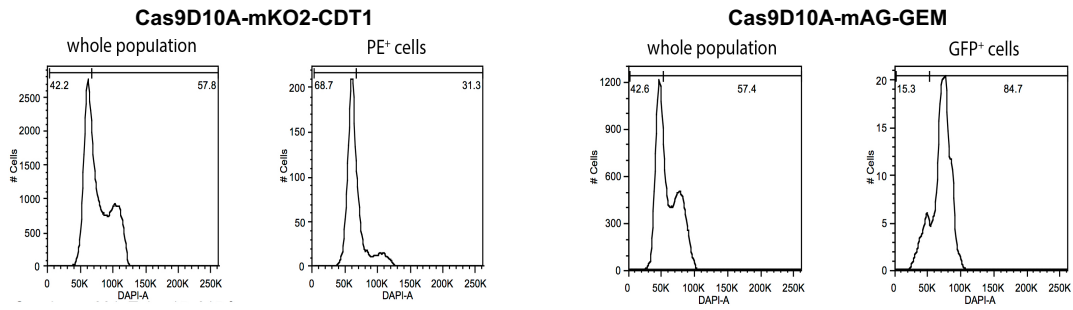

**B**

**anti-RPA1**

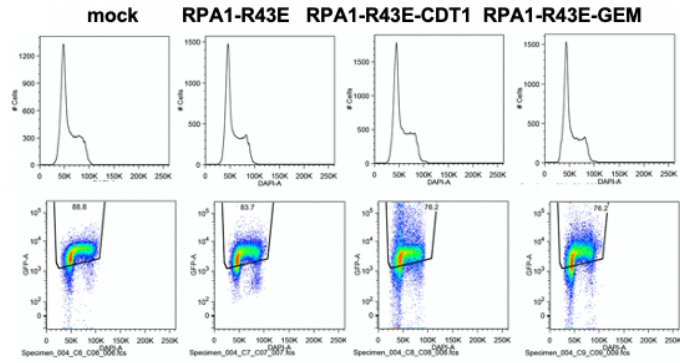

**anti-V5 tag**

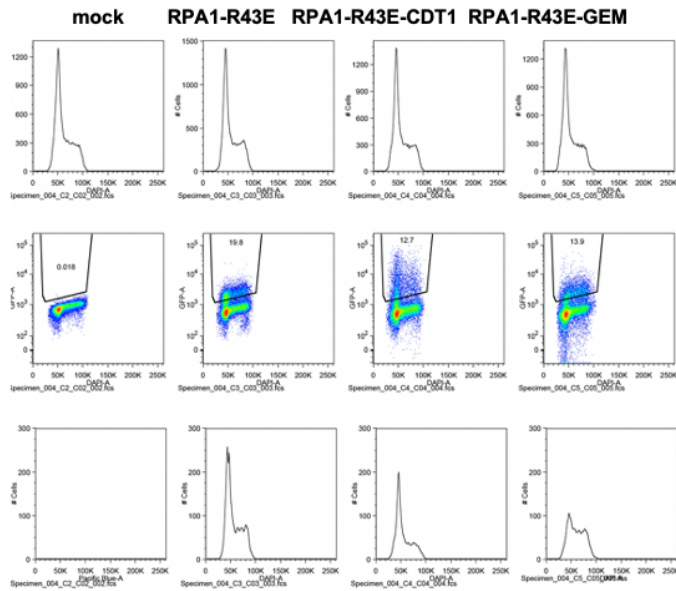

Supplement: S5 Fig — (A) Cell cycle profiles of 293T cells transfected with Cas9D10A-mKO2-CDT1 or Cas9D10A-mAGGEM expression constructs, showing either the entire population or the population gated for PE+ (mKO2) or GFP+ (mAG) cells. (B) Cell cycle profiles of cells mock-transfected or transfected with CDT1- or GEM-tagged derivatives of RPA1-R43E bearing a C-terminal V5 tag, and stained with anti-RPA antibody, which detects endogenous and ectopically expressed RPA; or with anti-V5 antibody, which detects ectopically expressed RPA1-R43E. (PDF) [file pgen.1009329.s005.pdf]
