# Supplementary material for: Pathways and signatures of mutagenesis at targeted DNA nicks
Source: PLoS Genet. 2021 Apr 15;17(4):e1009329. doi: 10.1371/journal.pgen.1009329 (PMC8078790; doi:10.1371/journal.pgen.1009329)

A

a-HDR: Nick/ssDNA		c-HDR: DSB/dsDNA	
**siRNA**	**HDR**	**siRNA**	**HDR**
siNT2	0.08	siNT2	1.0
siBRCA2	1.0	siBRCA2	0.08
siDNA2+siBRCA2	0.55	siDNA2	0.41
siEXO1+siBRCA2	1.0	siEXO1	0.91
siMRE11+siBRCA2	0.95	siMRE11	0.44

B

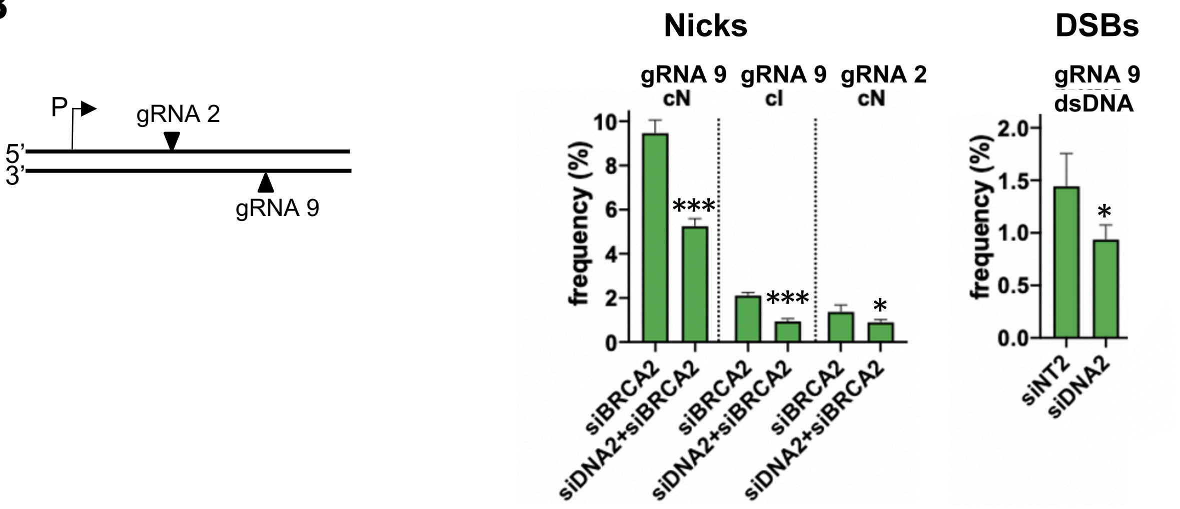

C

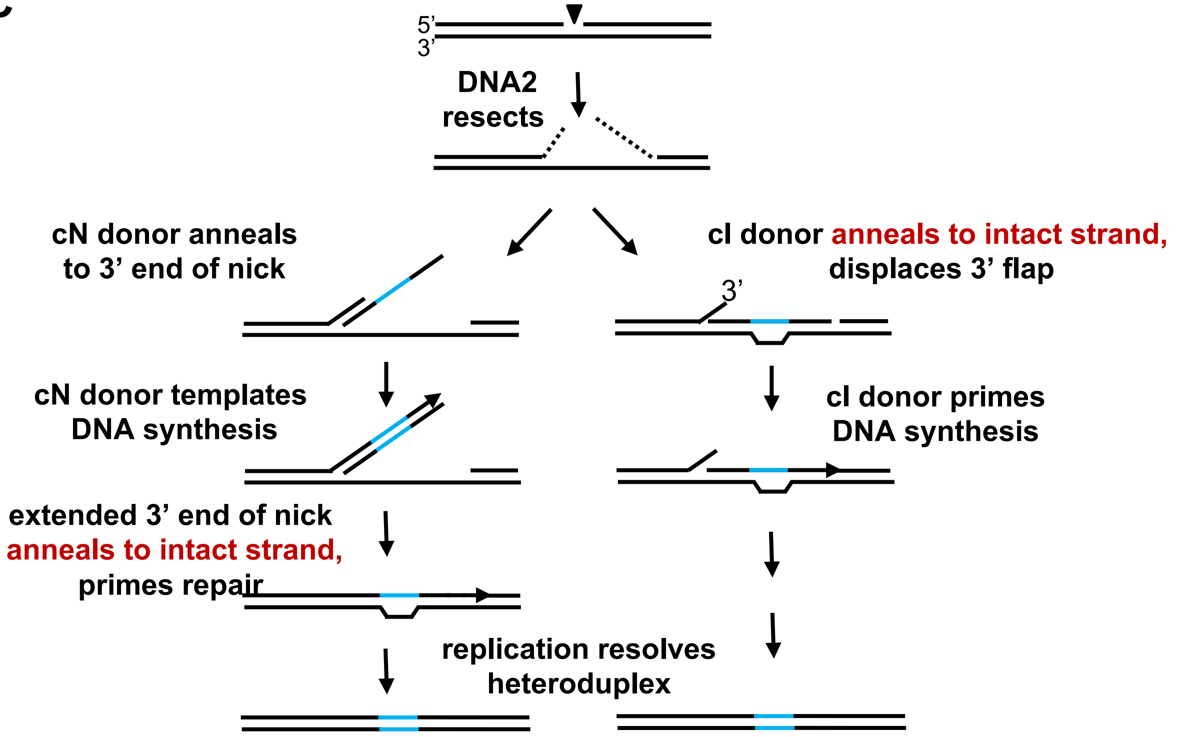

Supplement: S3 Fig — (A) Effects of DNA2, EXO1 or MRE11 depletion on frequencies of a-HDR or c-HDR at nicks or DSBs, respectively. Frequencies were normalized relative to frequencies in 293T TL cells treated with siBRCA2 (nicks) or siNT2 (DSBs). Cleavage was targeted by gRNA9 (see panel B) and supported by a cN ssDNA donor for a-HDR (nicks) or a plasmid donor for c-HDR (DSBs). (B) Left, diagram of the cleavage sites for gRNA 2 and gRNA 9 in the TL construct (arrowheads), with the promoter (P) upstream. Right, effects of depletion of DNA on frequencies of HDR at nicks or DSBs targeted to the TL reporter construct in 293T TL cells by the indicated gRNA. HDR was supported at nicks by a donor complementary to the nicked or intact strand (cN or cI, respectively), in cells treated with siNT2, siBRCA2 or siDNA2+siBRCA2, as indicated; or at DSBs by a dsDNA donor, in cells treated with siNT2 or siDNA2. Frequency values represent the mean ± SEM from a representative experiment; and * and *** indicate p<0.05 and p<0.001, respectively, for the frequency difference between indicated sample and sample treated with siBRCA2 (nicks) or siNT2 (DSBs). (C) Working model for the role of DNA2 resection in a-HDR at nicks supported by a cN or cI donor. Results in panel B show that DNA2 promotes a-HDR by both pathways, and the first step shown in resection 3’ of the nick. The cN or cI donors anneal as shown, and processing then generates a heteroduplex which is resolved by replication. (PDF) [file pgen.1009329.s003.pdf]
