# Supplementary material for: Pathways and signatures of mutagenesis at targeted DNA nicks
Source: PLoS Genet. 2021 Apr 15;17(4):e1009329. doi: 10.1371/journal.pgen.1009329 (PMC8078790; doi:10.1371/journal.pgen.1009329)
Supplement: S6 Fig — (A) Tabulated effects of depletion of indicated factors on frequencies of 1 bp insertions of A, C, G or T or all nucleotides at nicks targeted by gRNA 4. These and other sequences shown illustrate the top DNA strand, which is the strand targeted for nicks by gRNA 4/Cas9D10A (S1 Fig). (B) Graph of effects of depletion of indicated factors on frequency of molecules bearing +1bp insertions of A, C, G or T at the gRNA 4 target site. (C) Sequence of predominant 1 bp insertions at the gRNA 4 target site. Nick site, underlined; PAM, blue font; insertion, red font. (D) Tables show nucleotides identified at indicated positions in products of repair containing a 1 bp insertion within the 4 bp region spanning nick site, in U2OS cells treated as indicated Positions are numbered -2, -1, +1, +2, relative to the nick site in the reference sequence GT/GC, where the slash marks the site at which the nick targeted to the CD44 gene by gRNA 4 cleaves the phosphodiester backbone. (PDF) [file pgen.1009329.s006.pdf]

A

	1 bp insertions at nicks (frequency, %)				
siRNA	ALL	A	C	G	T
siNT2	0.40%	0.12%	0.01%	0.27%	0
siBRCA2	0.63%	0.05%	0.02%	0.55%	0.01%
siDNA2+siBRCA2	2.23%	0.11%	0.05%	2.11%	0
sREV1+siBRCA2	0.14%	0.03%	0.01%	0.1%	0.001%
siREV3+siBRCA2	2.16%	0.2%	0.09%	1.80%	0.07%
siREV7+siBRCA2	0.58%	0.03%	0.05%	0.49%	0.01%
siPOLQ+siBRCA2	0.78%	0.06%	0.01%	0.69%	0.02%

B

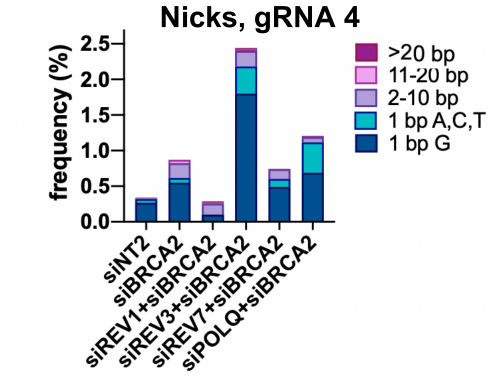

C

Nick site

5′CCTCGTGCCGCT
3′GGAGCACGGCGA

Predominant insertion

5′CCTCGTGGCCGCT
3′GGAGCACCGGCGA

D

siNT2		1 bp insertion frequency (%)			
position	ref	A	C	G	T
-2	G	0.001	0	0	0
-1	T	0.012	0.010	0.267	0
+1	G	0	0	0	0
+2	C	0	0	0	0

siBRCA2		1 bp insertion frequency (%)			
position	ref	A	C	G	T
-2	G	0	0	0	0.013
-1	T	0.046	0.023	0.549	0
+1	G	0	0.001	0	0
+2	C	0	0	0.001	0

siDNA2 +siBRCA2		1 bp insertion frequency (%)			
position	ref	A	C	G	T
-2	G	0	0	0	0.018
-1	T	0.111	0.012	2.108	0
+1	G	0.001	0	0	0
+2	C	0	0	0	0.016

siREV1 +siBRCA2		1 bp insertion frequency (%)			
position	ref	A	C	G	T
-2	G	0	0.010	0	0.001
-1	T	0.010	0.002	0.097	0
+1	G	0	0	0	0.001
+2	C	0.017	0	0	0

siREV3 +siBRCA2		1 bp insertion frequency (%)			
position	ref	A	C	G	T
-2	G	0.010	0	0	0.071
-1	T	0.195	0.085	1.800	0
+1	G	0	0	0	0.001
+2	C	0	0	0	0

siREV7 +siBRCA2		1 bp insertion frequency (%)			
position	ref	A	C	G	T
-2	G	0	0	0	0.007
-1	T	0.034	0.052	0.485	0
+1	G	0	0	0	0
+2	C	0	0	0	0.001

siPOLQ +siBRCA2		1 bp insertion frequency (%)			
position	ref	A	C	G	T
-2	G	0	0	0	0.017
-1	T	0.058	0.007	0.685	0
+1	G	0	0	0	0
+2	C	0	0	0	0
