# Supplementary material for: Pathways and signatures of mutagenesis at targeted DNA nicks
Source: PLoS Genet. 2021 Apr 15;17(4):e1009329. doi: 10.1371/journal.pgen.1009329 (PMC8078790; doi:10.1371/journal.pgen.1009329)

A

DSBs targeted by gRNA 4

	+1 bp insertions (frequency, %)			
**siRNA**	**A**	**C**	**G**	**T**
**siNT2**	2.16%	0.64%	17.9%	--
**siDNA2**	2.61%	0.93%	16.8%	0.70%
**siBRCA2**	2.03%	0.88%	16.8%	0.63%

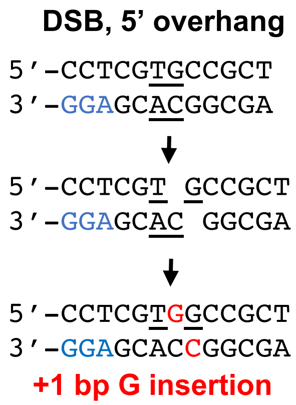

B

Nicks targeted by gRNA 7

	+1 bp insertions (frequency, %)			
**siRNA**	**A**	**C**	**G**	**T**
**siNT2**	0	0	0	0.33%
**siBRCA2**	0	0	0	0.59%
**siDNA2+siBRCA2**	0	0	0	0.51%
**siREV1+siBRCA2**	0	0	0	0.37%
**siREV3+siBRCA2**	0	0	0	0.59%
**siPOLQ+siBRCA2**	0	0	0	0.43%

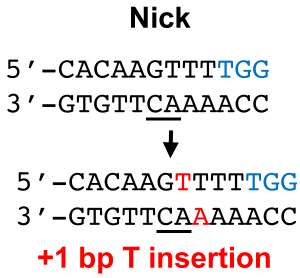

C

DSBs targeted by gRNA 7

	+1 bp insertions (frequency, %)			
**siRNA**	**A**	**C**	**G**	**T**
**siNT2**	0.38%	0.30%	0.39%	26.2%
**siDNA2**	0.57%	0.45%	0.53%	29.3%
**siBRCA2**	0.34%	0.33%	0.32%	18.2%

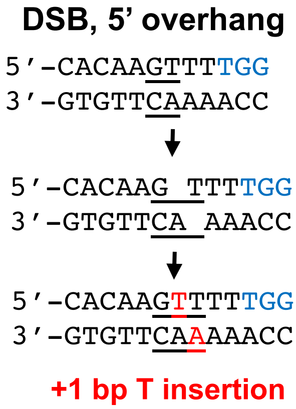

Supplement: S7 Fig — (A) Left, effects of depletion of indicated factors on frequencies of 1 bp insertions of A, C, G or T at DSBs targeted by gRNA 4. Right, the 12 bp region surrounding the gRNA 4 target site (underlined) at DSBs bearing 5’ overhangs; and +1G insertion at that site (red). PAM, blue font. The postulated repair intermediate is shown for clarity. (B) Left, effects of depletion of indicated factors on frequencies of 1 bp insertions of A, C, G or T at nicks targeted by gRNA 7. Right, the 12 bp region surrounding the gRNA 7 target site (underlined in nicked strand) and +1T insertion at that site (red). PAM, blue font. (C) Left, effects of depletion of indicated factors on frequencies of 1 bp insertions of A, C, G or T at nicks targeted by gRNA 7. Right, the 12 bp region surrounding the gRNA 7 target site (underlined) at DSBs bearing 5’ overhangs and +1T insertion at that site (red). PAM, blue font. The postulated repair intermediate is shown for clarity. (PDF) [file pgen.1009329.s007.pdf]
